# A heat-sensitive Osh protein controls PI4P polarity

**DOI:** 10.1186/s12915-020-0758-x

**Published:** 2020-03-13

**Authors:** Deike J. Omnus, Angela Cadou, Ffion B. Thomas, Jakob M. Bader, Nathaniel Soh, Gary H. C. Chung, Andrew N. Vaughan, Christopher J. Stefan

**Affiliations:** 1 MRC Laboratory for Molecular Cell Biology, University College London, Gower Street, London, WC1E 6BT United Kingdom; 2 Present address: Science for Life Laboratory, Department of Molecular Biosciences, The Wenner-Gren Institute, Stockholm University, Stockholm, Sweden; 3 Present address: Department of Proteomics and Signal Transduction, Max Planck Institute of Biochemistry, Martinsried, Germany

## Abstract

**Background:**

Phosphoinositide lipids provide spatial landmarks during polarized cell growth and migration. Yet how phosphoinositide gradients are oriented in response to extracellular cues and environmental conditions is not well understood. Here, we elucidate an unexpected mode of phosphatidylinositol 4-phosphate (PI4P) regulation in the control of polarized secretion.

**Results:**

We show that PI4P is highly enriched at the plasma membrane of growing daughter cells in budding yeast where polarized secretion occurs. However, upon heat stress conditions that redirect secretory traffic, PI4P rapidly increases at the plasma membrane in mother cells resulting in a more uniform PI4P distribution. Precise control of PI4P distribution is mediated through the Osh (oxysterol-binding protein homology) proteins that bind and present PI4P to a phosphoinositide phosphatase. Interestingly, Osh3 undergoes a phase transition upon heat stress conditions, resulting in intracellular aggregates and reduced cortical localization. Both the Osh3 GOLD and ORD domains are sufficient to form heat stress-induced aggregates, indicating that Osh3 is highly tuned to heat stress conditions. Upon loss of Osh3 function, the polarized distribution of both PI4P and the exocyst component Exo70 are impaired. Thus, an intrinsically heat stress-sensitive PI4P regulatory protein controls the spatial distribution of phosphoinositide lipid metabolism to direct secretory trafficking as needed.

**Conclusions:**

Our results suggest that control of PI4P metabolism by Osh proteins is a key determinant in the control of polarized growth and secretion.

**Electronic supplementary material:**

**Supplementary information** accompanies this paper at 10.1186/s12915-020-0758-x.

## Background

Phosphatidylinositol 4-phosphate (PI4P) is emerging as a key determinant of plasma membrane (PM) identity and function. PI4P has vital roles in the control of PM ion channels and the general recruitment of polybasic proteins to the PM [1]. PI4P may even serve as a spatial cue or signpost for protein targeting to specialized PM domains. For instance, PI4P is highly enriched in the primary cilium of neural progenitor cells [2, 3]. PI4P is also enriched at the growing tips of the pathogenic filamentous fungus *Candida albicans*, suggesting an evolutionarily conserved role in polarized growth [4]. In budding yeast, PI4P organizes the actin cytoskeleton and is proposed to target the p21-activated kinase Cla4 to sites of polarized growth [5, 6]. However, regulatory mechanisms that control PI4P distribution at the PM are not fully understood and are even controversial.

Synthesis of PI4P at the PM is carried out by phosphatidylinositol 4-kinase type IIIα (also known as PI4KIIIα), which is encoded by PI4KA in mammals and the *STT4* gene in *Saccharomyces cerevisiae* [5, 7, 8]. The yeast Stt4 PI4KIIIα protein localizes to cortical assemblies termed PIK (phosphoinositide kinase) patches, consistent with its essential role in generation of PI4P at the PM [5, 9, 10]. Curiously however, PI4P is enriched in the PM of growing daughter cells (buds) while cortical Stt4 PIK patch assemblies are found extensively in mother cells [9, 10]. It is unknown how PI4P accumulates in the growing bud and how PI4P levels are kept relatively low in mother cells where Stt4 PIK patches reside. Here, we address the paradoxical distribution between Stt4 PIK patches and their product PI4P. We find that Stt4 PIK patches are associated with the cortical endoplasmic reticulum (ER) in mother cells. Junctions between the ER and PM, also termed ER-PM contacts, are thought to be sites for PI4P-mediated non-vesicular lipid exchange reactions carried out by the conserved ORP/Osh protein family [11–14]. Accordingly, we find that members of the ORP/Osh protein family are necessary for proper distribution of PI4P at the PM.

Moreover, we show that changes in environmental conditions influence PI4P utilization and distribution. Upon heat shock conditions known to disrupt polarized secretion [15], non-vesicular PI4P consumption is attenuated, resulting in the generation of a PI4P signal in mother cells and reduced PI4P polarity. This control is achieved, at least in part, through inactivation of the Osh3 protein that extracts PI4P and delivers it to an ER-localized PI4P phosphatase. Interestingly, Osh3 is a heat stress-sensitive protein and rapidly forms intracellular aggregates upon heat shock. Notably, the PI4P-binding ORD region of the Osh3 protein is sufficient for aggregation, providing a rapid and direct mechanism for the regulation of PI4P as needed. We propose that the control of PI4P metabolism may provide a conserved mechanism to direct polarized growth and cellular responses to changes in environmental conditions.

## Results

### PI4P is polarized and regulated in response to physiological cues

**Fig. 1:**
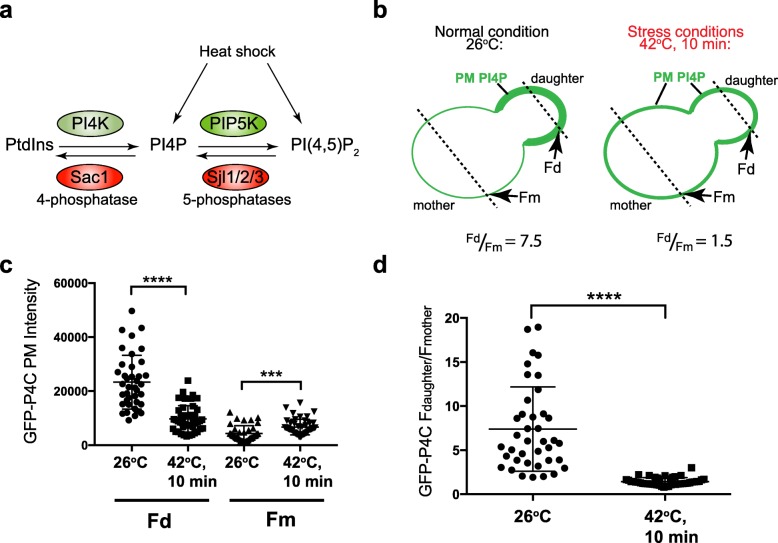
PI4P distribution is regulated by growth conditions. **a** Schematic representation of kinases and phosphatases involved in PI4P and PI(4,5)P_2_ metabolism. Heat shock elicits PI4P- and PI(4,5)P_2_-mediated signaling responses including regulated exocytosis and endocytosis. **b** Schematic representation of the method used to measure PM GFP-P4C fluorescence intensities at 26 °C and after 42 °C heat shock. Briefly, line scans were applied through both daughter and mother cells using Fiji, and the peak values corresponding to the GFP-P4C fluorescence intensity at the PM in the daughter (Fd) and mother cell (Fm) were recorded to calculate Fd/Fm ratios. **c** Quantitation of GFP-P4C fluorescence at the plasma membrane (PM) of daughter (Fd) and mother (Fm) cells at 26 °C and after a 10 min 42 °C heat shock. Graph shows the Fd and Fm mean values of individual cells. Total number of cells analyzed in three independent experiments: 26 °C, *n* = 40, 10 min 42 °C, *n* = 45. Error bars show standard deviations. The changes in Fd and Fm after heat shock are statistically significant (*t* test, ****p < 0.0001, ***p = 0.0002). **d** Quantitation of GFP-P4C fluorescence at the plasma membrane (PM) of daughter (Fd) and mother (Fm) cells at 26 °C and after a 10 min heat shock as described in **c**. Graph shows the Fd/Fm ratio of individual cells. Total number of cells analyzed in three independent experiments: 26 °C, *n* = 40, 10 min 42 °C, *n* = 45. Error bars show standard deviations. The decrease in the Fd/Fm ratio after heat shock is statistically significant (*t* test, ****p < 0.0001)

Phosphoinositide lipid metabolism at the plasma membrane (PM) is controlled by specific lipid kinases and phosphatases (Fig. 1a). Yet how cells generate discrete phosphoinositide signals at the PM in response to physiological stimuli is still poorly understood. The phosphoinositide isoform phosphatidylinositol 4-phosphate (PI4P) may play an especially important, but underappreciated, role in polarized cell growth and cell signaling. Previous studies have reported that PI4P is enriched in small (growing) daughter cells in budding yeast [16–19]. However, these studies described qualitative observations rather than quantitative measurements and used a reporter that binds both PI4P and PI(4,5)P_2_ [20]. We therefore monitored PI4P distribution at the PM by quantitative microscopy using a validated PI4P FLARE (fluorescent lipid-associated reporter), the P4C domain of SidC that specifically binds PI4P [21, 22] (Fig. 1 and Additional file 1: Figure S1). Because PI4P localizes to intracellular compartments as well as the PM, GFP-P4C intensities co-localized with the PM marker (mCherry-2xPH^PLCδ^) were specifically measured (see examples in Additional file 1: Figures S1a-b). Under non-stress growth conditions (26 °C), the PI4P FLARE was highly enriched at the PM of daughter cells compared to mother cells (> 5-fold, Fig. 1b, c, Additional file 2: Fig. 1c and 1d Dataset). This polarized distribution suggests that PI4P may serve as a landmark at the PM, as has been proposed for other negatively charged lipids including PI(4,5)P_2_ and phosphatidylserine [1, 23–25].

If PI4P serves as a spatial landmark at the PM, then its distribution should change in response to physiological stimuli that regulate cell polarity. Heat elicits several responses in budding yeast cells including reorganization of the actin cytoskeleton, Rho GTPase signaling, and increased vesicle trafficking events in mother cells [9, 15, 26]. Upon brief heat shock (10 min at 42 °C), the polarized enrichment of PI4P in daughter cells was significantly reduced. There was a twofold decrease in GFP-P4C intensity at the PM in daughter cells (Fig. 1c and Additional file 2: Fig. 1c and d Dataset) and a twofold increase at the PM in mother cells (Fig. 1c, Additional file 1: Figure S1d, Additional file 2: Fig. 1c and d Dataset, and Additional file 3: Fig. S1c and S1d Dataset). Consequently, the ratio of PI4P reporter distribution between daughter and mother cells decreased significantly (fivefold) following heat shock (*F*_d_/*F*_m_ = 7.5 at 26 °C versus *F*_d_/*F*_m_ = 1.5 at 42 °C; Fig. 1b–d and Additional file 2: Fig. 1c and d Dataset). These responses occurred very rapidly, within 2–4 min (Additional file 1: Figures S1b-d and Additional file 3: Fig. S1c and S1d Dataset), suggesting a highly sensitive regulatory system.

**Fig. 2:**
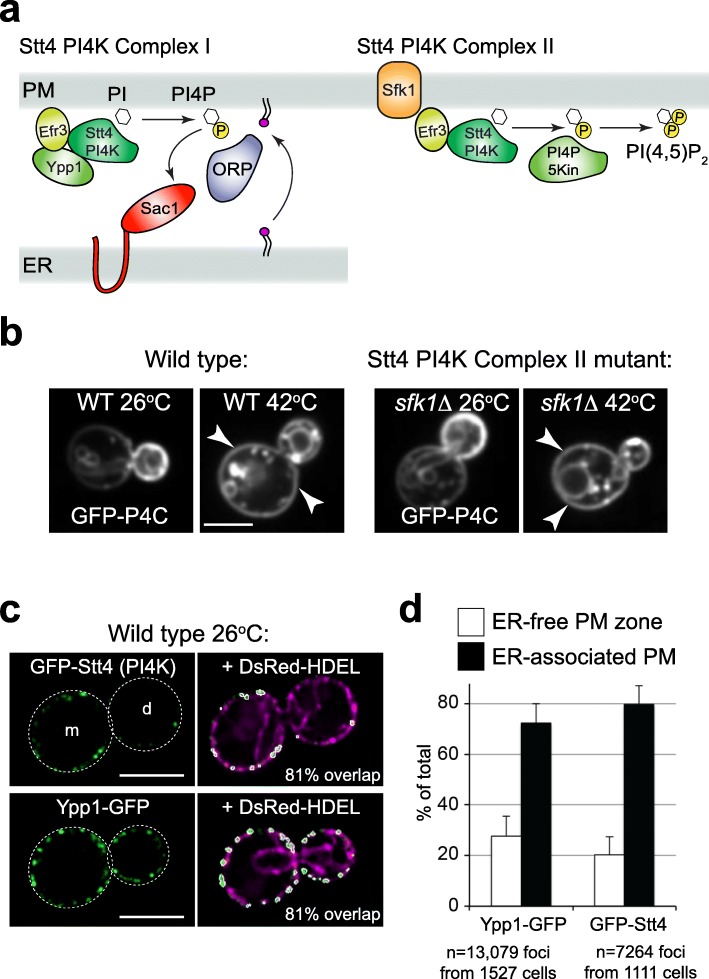
Stt4 PIK patches localize to ER-PM contact sites. **a** Schematic representation of two Stt4 PI 4-kinase complexes present at the PM. Stt4 complex I (Stt4/Efr3/Ypp1), also known as a PIK patch, is enriched in mother cells and is essential for Osh3 PM localization. ORP/Osh protein family members are proposed to mediate lipid transfer and exchange at ER-PM contacts resulting in PI4P consumption by the Sac1 phosphatase. Complex II (Stt4/Efr3/Sfk1) is involved in stimulus-induced PI4P and PI(4,5)P_2_ synthesis. **b** Sfk1 (Stt4 Complex II) is not required for heat-induced PI4P signaling in mother cells. GFP-P4C fluorescence indicating PI4P localization in wild type (left panels) and *sfk1*Δ cells (right panels) at 26 °C and 42 °C. Arrows point out increased PI4P levels at the PM of mother cells. Scale bar, 5 μm. **c** Stt4 complex I (PIK patches) are ER-associated. Wild type cells expressing GFP-Stt4 or Ypp1-GFP (green) co-expressed the ER marker DsRed-HDEL (magenta). Mother (m) and daughter (d) cells are indicated (dashed). Stt4- and Ypp1-containing PIK patches (outlined in white) were automatically segmented and scored for co-localization with the ER marker. Scale bar, 4 μm. **d** High-content quantitative analysis of PIK patch localization with the ER. Maxima from GFP-Stt4 puncta (7264 from 1111 cells) and Ypp1-GFP puncta (13,079 from 1527 cells) were identified using Fiji. Maxima positive for the ER marker DsRed-HDEL were scored as ER-associated. Results show the mean and standard deviation from three independent experiments

We next investigated potential mechanisms for the increase in the PI4P signal at the PM in mother cells that could result from increased synthesis and/or decreased hydrolysis. PI4P is generated at the PM in budding yeast by the Stt4 protein, an ortholog of phosphatidylinositol 4-kinase type IIIα (PI4KIIIα) [5]. Accordingly, GFP-P4C is lost from the PM in temperature conditional *stt4* mutant cells following heat shock (Additional file 4: Figure S2a). PI4KIIIα is present in two distinct protein complexes. PI4KIIIα complex I is comprised of Stt4/Efr3/Ypp1 in yeast and PI4KIIIα/EFR3/TTC7/FAM126A in mammalian cells (Fig. 2a) [8, 10, 27]. PI4KIIIα complex II consists of Stt4/Efr3/Sfk1 in yeast and PI4KIIIα/EFR3/TMEM150 in mammalian cells (Fig. 2a) [9, 28]. Sfk1 is necessary for heat-induced PI(4,5)P_2_ synthesis in yeast and the TMEM150 proteins are involved in PI(4,5)P_2_ re-synthesis following phospholipase C activation [9, 28]. We tested if the heat-induced increase in PI4P at the PM in mother cells required the “signaling” Stt4 PI4K complex II implicated in heat-induced PI(4,5)P_2_ synthesis. Surprisingly, heat shock induced a PI4P signal at the PM in mother cells lacking Sfk1 (Fig. 2b). Thus, the Sfk1-containing Stt4 PI4K complex II is not absolutely required for heat-induced PI4P redistribution from daughter cells to mother cells. The Stt4 PI4K complex I may then also contribute to the inducible PI4P signal in mother cells. Accordingly, heat-induced GFP-P4C redistribution was impaired, but not completely blocked, in *ypp1–*7 mutant cells (Additional file 4: Figure S2b and Additional file 5: Fig. S2b Dataset). In support of these results, previous work has indicated that Ypp1 is involved in the bulk of PI4P synthesis at the PM [10, 29]. Moreover, a recent study has implicated PI4K complex I in stimulus-induced PI(4,5)P_2_ synthesis in metazoan cells [30]. Thus, both Stt4 PI4K complexes may be involved in generation of the heat-induced PI4P signal in mother cells.

### Stt4 PI4K assemblies localize to ER-PM contacts

Cortical Stt4 PI4K assemblies—also termed PIK patches—are enriched in mother cells even under non-stress growth conditions [9, 10] (Fig. 2c). This is surprising in light of the finding that PI4P is enriched at the PM of daughter cells, not mother cells, under non-stress growth conditions (Fig. 1 and Additional file 1: Figure S1). It is proposed that Stt4 PIK patches may reside at contacts between the PM and ER [10], and ER-PM contacts have been implicated in PI4P regulation [16]. However, Stt4 localization at ER-PM contacts has not yet been demonstrated. We therefore investigated potential regulatory mechanisms for Stt4-generated PI4P by examining PIK patch localization in more detail. We used high-content quantitative microscopy to examine PIK patch distribution in cells co-expressing a GFP-tagged PIK patch protein (Stt4 or Ypp1) and an ER marker (DsRed-HDEL). PIK patches were automatically segmented and maxima for GFP signal intensities within individual PIK patches were subsequently identified. Next, the intensity of the DsRed-HDEL (ER) signal within each GFP maxima (PIK patch) was quantified. The dynamic range of DsRed-HDEL intensities in the ER was also measured to define a minimum specific level for the DsRed-HDEL ER signal and maximal non-specific background DsRed-HDEL levels in ER-free regions. These measurements set a threshold value to assign individual GFP maxima as either ER-associated or ER-free.

Stt4- and Ypp1-containing PIK patches extensively coincided with the peripheral ER (Fig. 2c, white traces show segmented GFP-Stt4 and Ypp1-GFP PIK patches; > 80% overlap with DsRed-HDEL in the examples shown, Additional file 6: Fig. 2c and S2c Dataset). The DsRed-HDEL ER marker overlapped with 80% of GFP-Stt4 patches identified in high-content experiments (7264 maxima from 1111 cells in three independent experiments, Fig. 2d and Additional file 7: Fig. 2d Dataset). Likewise, 76% of Ypp1-GFP cortical patches analyzed (13,079 maxima from 1527 cells) were ER-associated (Fig. 2d and Additional file 7: Fig. 2d Dataset). Loss of the reticulon proteins (Rtn1, Rtn2, and Yop1) that shape the ER network into highly curved tubules and membrane sheets with curved edges [31] did not alter PIK patch association with the cortical ER. GFP-Stt4 remained extensively associated with cortical ER sheets (78% overlap), and GFP-Stt4 was notably absent from regions of the PM lacking cortical ER in the reticulon mutant cells (*rtn1*∆ *rtn2*∆ *yop1*∆ mutant cells, Additional file 4: Figure S2c and Additional file 6: Fig. 2c and S2c Dataset). In contrast, localization of PIK patches with the ER depended on ER-PM contact formation. In cells lacking the ER-localized Scs2 and Scs22 proteins that form ER-PM contacts [16, 32], GFP-Stt4 patches were clearly observed at regions of the PM lacking cortical ER (*scs2*∆ *scs22*∆ mutant cells, Additional file 4: Figure S2c). Thus, Stt4 PI4K complex I assemblies (PIK patches) are extensively associated with the cortical ER and this arrangement may impact PI4P metabolism in mother cells.

### PM-localized PIK patches are in proximity to Scs2 and the Sac1 PI4P phosphatase in the cortical ER

**Fig. 3:**
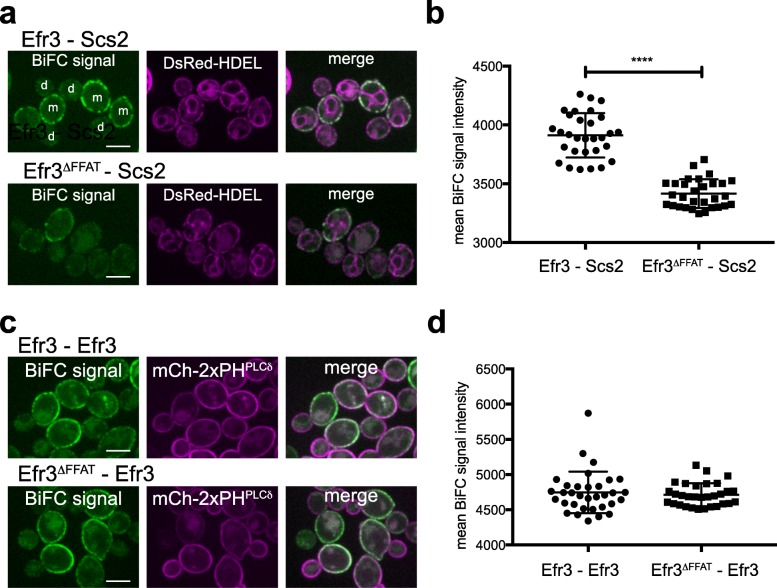
The PIK patch subunit Efr3 interacts with ER-localized Scs2. **a** The Efr3 FFAT motif promotes Scs2-Efr3 interactions. Interactions between Scs2 and Efr3 or Efr3^∆FFAT^ as scored by BiFC. Cells expressed Efr3-GFP_N_ (upper panel) or Efr3^∆FFAT^-GFP_N_ (lower panel) along with GFP_C_-Scs2 and the ER marker DsRed-HDEL (magenta). Scale bars, 5 μm. **b** Quantitation of specific BiFC signals generated by interaction of Efr3-GFP_N_ or Efr3^∆FFAT^-GFP_N_ with GFP_C_-Scs2 at the ER using DsRed-HDEL to define regions of interest. Graph shows the mean values and standard deviations from three independent experiments. Each point represents the mean of an image frame (with > 10 cells/frame); 30 frames (> 300 cells) were analyzed for each. The difference between the BiFC signal in cells expressing Efr3-GFP_N_ versus Efr3^∆FFAT^-GFP_N_ with GFP_C_-Scs2 was statistically significant (t test, ****p < 0.0001). **c** The Efr3 FFAT motif is not required for Efr3-Efr3 interactions. Cells expressed Efr3-GFP_N_ (upper panel) or Efr3^∆FFAT^-GFP_N_ (lower panel) together with Efr3-GFP_C_ and the PM marker mCherry-2xPH^PLCδ^. Scale bars, 5 μm. **d** Quantitation of specific BiFC signals generated by interaction of Efr3-GFP_N_ or Efr3^∆FFAT^-GFP_N_ with Efr3-GFPc using mCherry-2xPH^PLCδ^ to specify Efr3-Efr3 interactions at the PM. Graph shows the means and standard deviations from three independent experiments. Each point represents the mean value from an image frame (> 10 cells/frame). Total number of frames: Efr3–Efr3 *n* = 33, Efr3^∆FFAT^–Efr3 *n* = 31. The results show no difference between Efr3-GFP_N_ versus Efr3^∆FFAT^-GFP_N_ in association with Efr3-GFPc

We investigated potential associations between Stt4 PI4K complex I subunits and Scs2 in further detail. Scs2, a VAP-A/B ortholog, is a tail-anchored ER membrane protein with an N-terminal cytoplasmic MSP domain. The MSP domain binds proteins with a FFAT motif (two phenylalanines in an acidic tract) [33]. Examination of Stt4 PI4K complex I proteins with an algorithm that scores FFAT motifs [33] revealed a candidate FFAT-like motif in the C-terminus of the Efr3 protein (… GENQN**DDFKDANED**LHSLSSRGKIFSST_782_). We employed bimolecular fluorescence complementation (BiFC or split GFP; Fig. 3 and Additional file 8: Figure S3) assays to address whether the ER-localized Scs2 protein is in proximity to the PM-localized Efr3 protein. Cortical GFP patches were observed in cells co-expressing Efr3-GFP_N_ and GFP_C_-Scs2 fusions (Fig. 3a). Interestingly, the Efr3-Scs2 interaction occurred in mother cells but not daughter cells, and at cortical ER structures but not in cytoplasmic ER or nuclear ER regions (Fig. 3a). The FFAT motif in Efr3 was necessary for efficient association with Scs2, as the split GFP signal intensity significantly decreased in cells expressing a truncated Efr3^∆FFAT^-GFP_N_ fusion and GFP_C_-Scs2 (Fig. 3a, b and Additional file 9: Fig. 3b Dataset). In control experiments, the full-length Efr3-GFP_N_ and truncated Efr3^∆FFAT^-GFP_N_ fusions both interacted with Efr3-GFP_C_, indicating that the truncated form of Efr3 lacking the FFAT motif was expressed (Fig. 3c, d and Additional file 10: Fig. 3d Dataset). In addition to Scs2, Efr3 was in proximity to the ER-localized PI4P phosphatase Sac1 as assessed by BiFC (Additional file 8: Figure S3b). Similar to Efr3, Sac1 displayed homotypic interactions at cortical patches (Additional file 8: Figure S3b), suggesting Sac1 may be clustered in the cortical ER. In additional control experiments, Efr3 interacted with Ypp1 and Sac1 interacted with Scs2 by BiFC (Additional file 8: Figure S3b), consistent with published data [10, 16, 34]. Thus, ER-localized Scs2 is in proximity to the Stt4 PIK patch subunit Efr3 at the PM and interacts with the Sac1 PI4P phosphatase in the ER. This configuration may allow for the efficient control of PI4P generated by Stt4 at the PM in mother cells.

### Osh3-mediated PI4P hydrolysis is attenuated during heat shock

Our results suggest that Stt4 PIK patches localize to ER-PM contacts (Figs. 2 and 3) that are implicated in PI4P regulation [16]. Upon heat shock, ER-PM contacts may disassemble or Stt4 may move to ER-free PM zones, impairing Sac1-mediated PI4P hydrolysis in mother cells. However, cortical ER structures are observed and Stt4 PIK patches remain extensively co-localized with the cortical ER network during heat shock (Additional file 11: Figure S4a-b). We then further investigated how heat-induced PI4P signaling at the PM of mother cells is triggered.

**Fig. 4:**
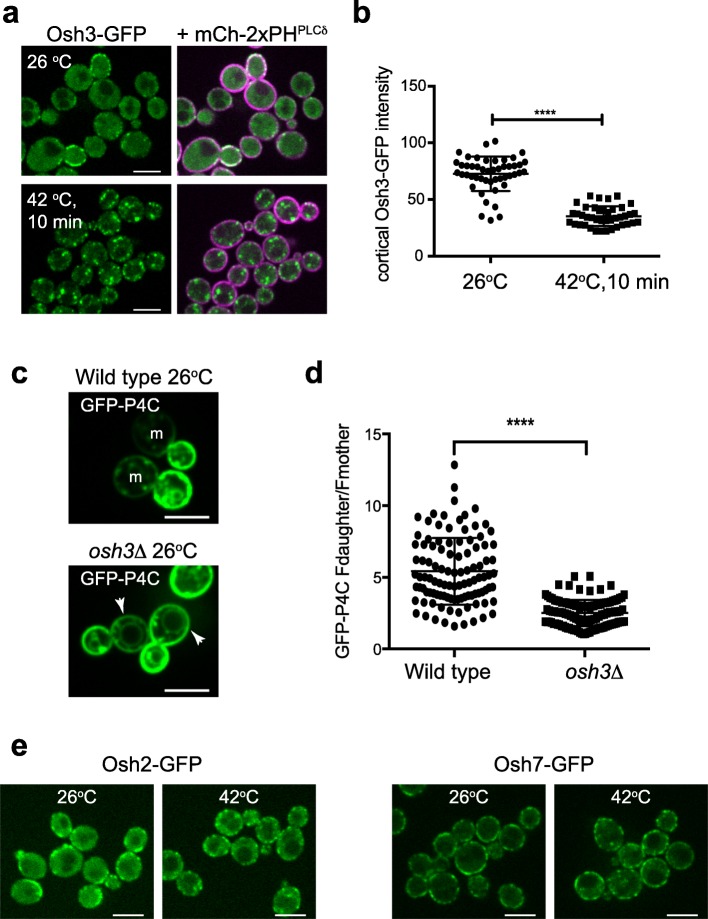
Osh3 regulates PI4P metabolism and distribution. **a** Localization of Osh3-GFP (green) and the PM marker mCherry-2xPH^PLCδ^ (magenta) at 26 °C and after 10 min heat shock at 42 °C. Upon heat shock, Osh3 switches from cortical patches to internal puncta. Scale bars, 5 μm. **b** Quantitation of cortical Osh3-GFP fluorescence intensity in cells at 26 °C versus 10 min 42 °C heat shock. The PM marker mCherry-2xPH^PLCδ^ was used to define cortical regions of interest. Graph shows the means and standard deviations from three independent experiments. Points present mean values of individual image frames (> 10 cells/frame). Total number of frames analyzed: 26 °C *n* = 48, 42 °C *n* = 41. The decrease of cortical Osh3-GFP fluorescence after heat shock was statistically significant (*t* test, ****p < 0.0001). **c** Wild type (upper panel) and *osh3*∆ (lower panel) cells expressing the PI4P reporter GFP-P4C grown at 26 °C. Arrows point to PI4P at the PM in mother (m) cells. Scale bars, 5 μm. **d** Quantitation of cortical GFP-P4C fluorescence intensity in wild type and *osh3*∆ cells grown at 26 °C. See Fig. 1c for details of analysis. Graph shows the Fd/Fm ratios of individual cells from three independent experiments, bars represent mean and standard deviations (*t* test, ****p < 0.0001). Number of cells analyzed: WT *n* = 105, *osh3*∆ *n* = 117. **e** Cells expressing Osh2-GFP (left panel) or Osh7-GFP (right panel) grown at 26 °C and subjected to a heat shock for 10 min at 42 °C. Scale bars, 5 μm

Scs2 binds and recruits the FFAT motif-containing Osh3 protein [19, 35]. Osh3 is a member of a conserved family of lipid exchange proteins, the oxysterol-binding protein related proteins (ORP). The yeast Osh2 and Osh6/7 proteins bind ergosterol and phosphatidylserine, respectively, in vitro and are proposed to transfer these lipids from the ER to the PM in exchange for PI4P [12, 13] (Fig. 2a). It is currently not established if Osh3 is a lipid transfer protein, but Osh3 has been shown to bind PI4P and to activate the Sac1 PI4P phosphatase in vitro [19, 36]. We examined whether Osh proteins implicated in metabolism of PM PI4P pools (by extracting PI4P and delivering PI4P to the ER in exchange for another lipid or directly presenting PI4P to the Sac1 phosphatase) might be attenuated during heat shock. Under normal growth conditions, Osh3-GFP is observed at cortical patches as well as diffusely localized in the cytoplasm (Fig. 4a). Upon heat shock, cortical localization of Osh3-GFP (cells co-expressed the PM marker mCherry-2xPH^PLCδ^) was significantly reduced and instead Osh3-GFP localized to numerous intracellular puncta (Fig. 4a, b and Additional file 12: Fig. 4b Dataset).

We also addressed whether inactivation of Osh3 may be involved in the heat-induced PI4P signal in mother cells. In support of this, the polarized distribution of PI4P was significantly reduced in *osh3*∆ cells, as GFP-P4C was increased at the PM in mother cells lacking Osh3, even under non-stress conditions (Fig. 4c). Accordingly, the ratio of PI4P distribution between daughter and mother cells was significantly decreased in cells lacking Osh3 (F_d_/F_m_ = 5.4 in wild type cells at 26 °C versus F_d_/F_m_ = 2.5 in *osh3*∆ cells at 26 °C; Fig. 4d and Additional file 13: Fig. 4d Dataset). Additional Osh proteins that localize to ER-PM contacts (Osh2, Osh6, and Osh7) [13] also contribute to PI4P polarization, as localization of the PI4P reporter increased in mother cells lacking these proteins (Additional file 11: Figure S4c). Osh2 and Osh7 remained at cortical patches upon heat shock (Fig. 4e), although the results do not address whether these proteins are attenuated upon heat shock. Altogether, the results show that Osh proteins are important for polarized PI4P distribution under non-stress conditions and suggest that inactivation of Osh3 may be involved in the generation of heat-induced PI4P signals in mother cells.

### The Osh3 protein undergoes heat-induced aggregation

**Fig. 5:**
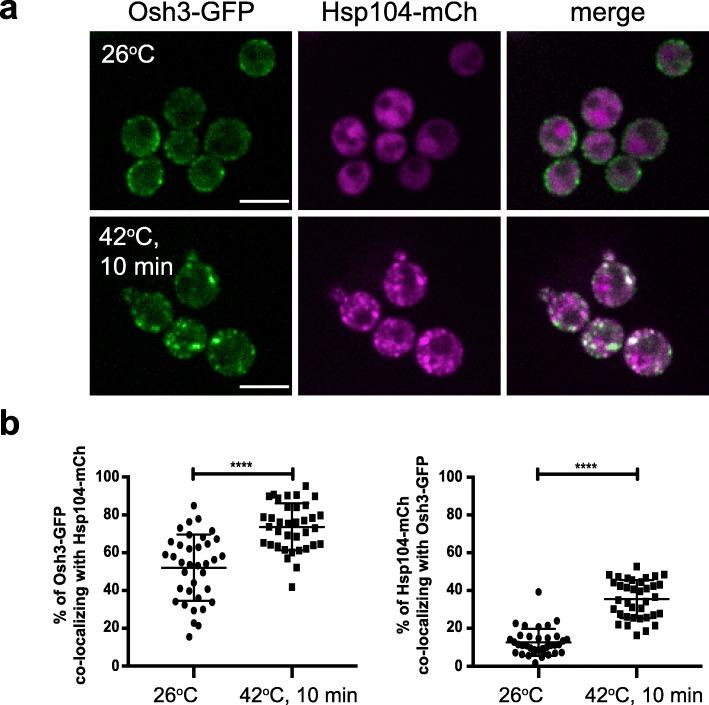
Osh3 co-localizes with Hsp104 under heat stress conditions. **a** Cells expressing Osh3-GFP (green) and Hsp104-mCherry (magenta) grown at 26 °C (upper panel) and after a 10 min 42 °C heat shock (lower panel). Scale bar, 5 μm. **b** Quantitation of Osh3-GFP and Hsp104-mCherry co-localization at 26 °C and 42 °C heat shock. Graphs show the means and standard deviations from three independent experiments (*t* test, ****p < 0.0001). The points present values of individual image frames (> 10 cells/frame). Number of frames analyzed: 26 °C *n* = 36, 42 °C *n* = 36

We further examined the intracellular Osh3 puncta formed during heat shock. Osh3-GFP extensively localized with Hsp104 upon heat shock (Fig. 5a, b, Additional file 14: Fig. 5b Dataset). Hsp104 is a disaggregase that functions to refold denatured proteins upon stress conditions including heat shock [37]. Thus, Osh3 may undergo a phase transition and aggregate upon heat stress conditions. Accordingly, the heat-induced Osh3-GFP structures did not extensively co-localize with Golgi compartments, endosomes, the ER, or lipid droplets (Additional file 15: Figure S5a). In additional control experiments, GFP localized diffusely throughout the cytoplasm and did not form intracellular aggregates upon heat shock (Additional file 15: Figure S5b).

**Fig. 6:**
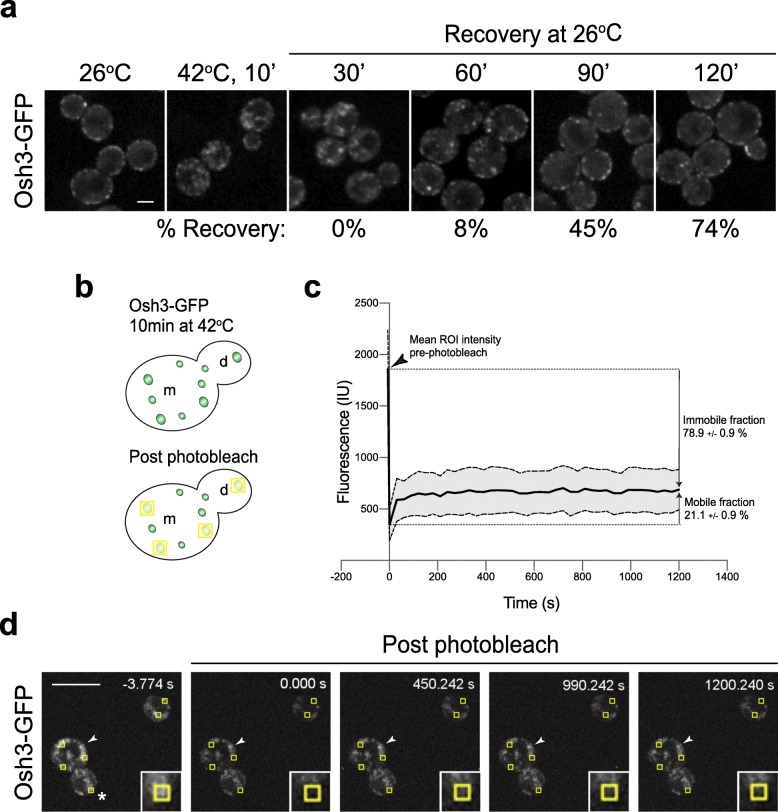
Osh3 aggregates under heat stress conditions. **a** Recovery of Osh3-GFP to the plasma membrane after heat stress and return to non-stress conditions. Exponentially growing wild type cells expressing Osh3-GFP were grown at 26 °C, shifted to 42 °C for 10 min, and allowed to recover at 26 °C. Images were collected at the time points indicated. The percentage of cells without cytosolic aggregates at each time point is shown (averages of > 390 cells/time point from two independent experiments). Scale bar, 2 μm. **b** Schematic displaying fluorescence recovery after photobleaching (FRAP) analyses on Osh3-GFP intracellular puncta formed upon heat shock. The yellow boxes mark the photobleached regions. **c** Quantitative FRAP analyses of internal Osh3-GFP puncta formed after brief heat shock. The mean intensity of 22 puncta (ROI) from two independent experiments is shown prior to (arrowhead) and following photobleaching (solid black line). Standard deviation is shown (gray area between dashed lines). The mobile and immobile fractions are indicated. **d** Time-lapse images of cells with internal Osh3-GFP puncta formed after brief heat shock. Cells expressing Osh3-GFP were grown at 26 °C and shifted to 42 °C for 10 min prior to being placed on a coverslip for time-lapse FRAP imaging. Images were taken at the time points indicated. The yellow boxes demarcate the photobleached regions of interest (ROI) and the inset shows a × 3 magnification of an individual photobleached ROI (asterisk). The arrowheads indicate an example of an Osh3-GFP aggregate that was not photobleached and remained stable throughout the course of the experiment. Scale bar, 10 μm

We performed additional in vivo tests to characterize the material state of the heat-induced Osh3 puncta to determine whether they were phase-separated liquid droplets or stable protein aggregates. Once formed, the heat-induced Osh3 puncta were stable and persisted for more than 60 min following return to the non-stress temperature (26 °C, Fig. 6a, Additional file 16: Fig. 6a Dataset). Cortical Osh3 assemblies re-appeared 90–120 min following shift to the non-stress temperature, coinciding with the previously reported duration for budding yeast to adapt to heat stress and resume polarized growth [15]. We also performed in vivo FRAP (fluorescence recovery after photobleaching) experiments to characterize the material state of the heat-induced Osh3 structures (Fig. 6b–d). Phase-separated liquid condensates (liquid-liquid droplets) tend to undergo dynamic exchange and rapidly recover during FRAP assays, whereas stable protein aggregates do not display rapid recovery rates in FRAP experiments [38–40]. The heat-induced Osh3-GFP puncta displayed very modest recovery post photobleaching (approximately 20% of pre-photobleach levels, Fig. 6c, d, Additional file 17: Fig. 6c Dataset). Moreover, stable puncta were observed over the course of the entire experiment (20 min, Fig. 6d). Thus, Osh3 is a heat-sensitive protein that aggregates and co-localizes with the disaggregase Hsp104 upon heat stress conditions.

Surprisingly, Osh3 aggregation did not require Hsp104 or the Hsp42 proteins (Additional file 18: Osh3 Localization Dataset) implicated in the formation of Hsp104-containing Q-bodies [41]. Heat-induced Osh3 aggregation also occurred independently of numerous stress-activated factors (Additional file 18: Osh3 Localization Dataset), including Rsp5 E3 ubiquitin ligase activity and the heat-responsive protein kinases Pkc1 and Tor2 [15, 17]. This led us to ask if some intrinsic property of the Osh3 protein itself triggers heat-induced aggregation and we next investigated the relevant Osh3 protein domains. A distinguishing feature of the Osh3 protein is an N-terminal GOLD domain (Additional file 19: Figure S6). The GOLD domain was sufficient for heat-induced aggregation. Under non-stress conditions, a GOLD-GFP fusion localized diffusely throughout the cytoplasm, but the GOLD-GFP fusion protein formed intracellular puncta upon a brief heat shock (42 °C, 10 min; Additional file 19: Figure S6a). Unexpectedly however, the GOLD domain was not required for heat-induced Osh3 aggregation in vivo. An N-terminal truncation revealed that the C-terminal PI4P-binding ORD region was also sufficient for heat-induced aggregation. Under non-stress conditions, the ORD^Osh3^-GFP fusion protein localized diffusely in the cytoplasm and nucleus, but it formed intracellular puncta upon brief heat shock at 42 °C (Additional file 19: Figure S6b). The ORD domain was particularly heat-sensitive, as ORD^Osh3^-GFP and full-length Osh3-GFP formed aggregates even upon a brief shift to 37 °C (Additional file 19: Figure S6b). The ORD domain was required for PI4P polarization, as GFP-P4C localization was increased at the PM in mother cells expressing a truncated form of Osh3, even under non-stress conditions (Additional file 19: Figure S6c). Thus, the PI4P-binding ORD region of the Osh3 protein undergoes heat-induced aggregation and is required for PI4P regulation in mother cells.

**Fig. 7:**
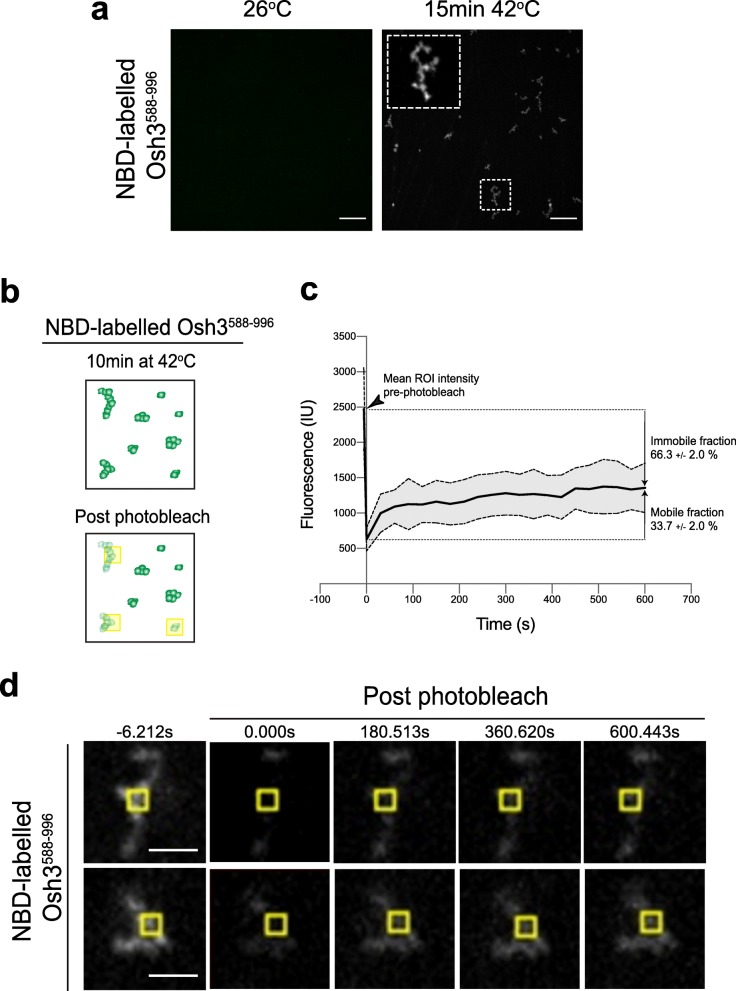
The Osh3 PI4P-binding ORD region forms condensates in vitro. **a** Purified NBD-labeled Osh3^588–996^ was imaged at 26 °C and after incubation at 42 °C. Upon elevated temperature, NBD-labeled Osh3^588–996^ assembles into visible condensates. The inset shows a × 5 magnification of an example of an Osh3^588–996^ condensate. Scale bars, 10 μm. **b** Scheme displaying fluorescence recovery after photobleaching (FRAP) analyses on NBD-labeled Osh3^588–996^ condensates formed after a 15-min incubation at 42 °C. The yellow boxes mark the regions subjected to five sequential 200 ms rounds of photobleaching. Fluorescence intensity decreased throughout the condensates upon photobleaching (see Fig. 6d for examples). **c** Quantitative FRAP analyses of NBD-labeled Osh3^588–996^ condensates formed after incubation at 42 °C. The mean intensity of regions of interest (ROI) from 8 condensates from two independent experiments is shown prior to (arrowhead) and following photobleaching (solid black line). Standard deviation is shown (gray area between dashed lines). The mobile and immobile fractions are indicated. **d** NBD-labeled Osh3^588–996^ condensates formed after incubation at 42 °C were placed on a coverslip for time-lapse FRAP imaging. Images were taken at the time points indicated. Images show individual NBD-labeled Osh3^588–996^ condensates and the yellow partitions demarcate the photobleached regions of interest (ROI). Scale bars, 2 μm

Consistent with the in vivo results, the purified Osh3 ORD region (Osh3^588–996^) displayed heat-sensitive properties in vitro. NBD-labeled Osh3^588–996^ protein formed punctate and large fibril-like structures upon a brief incubation at 42 °C (Fig. 7a). The structures were non-uniform in shape and size and did not display the typical round shape expected for a phase-separated liquid-liquid droplet (Fig. 7a) [38–40]. However, the heat-induced NBD-labeled Osh3^588–996^ structures displayed partial recovery in FRAP experiments (mobile fraction 30–35%; Fig. 7b–d and Additional file 20: Fig. 7c Dataset). In addition, successive rounds of UV excitation were required for efficient photobleaching and resulted in fluorescence intensity decreases outside the targeted region (Fig. 7d). These results suggest that the purified Osh3 ORD may form polymers with gel-like properties at elevated temperature. In sedimentation assays, purified Osh3^588–996^ was present in the soluble supernatant fraction at 26 °C, but efficiently pelleted upon brief incubation at 42 °C (Additional file 21: Figure S7a and Additional file 22: Fig. S7a Dataset). Likewise, NBD-labeled Osh3^588–996^ was present in the soluble supernatant fraction at 26 °C, but efficiently pelleted upon brief incubation at 42 °C (Additional file 21: Figure S7b). Thus, the Osh3 protein displays heat sensitivity in vitro and aggregates in vivo upon heat stress via its GOLD and ORD domains. Interestingly, additional Osh proteins displayed sensitivity to heat in vitro. In sedimentation assays, Osh4, Osh6, and Osh7 (which primarily consist of an ORD domain) were predominantly present in the soluble fraction at 26 °C but pelleted upon incubation at 42 °C (Additional file 21: Figure S7a and Additional file 22: Fig. S7a Dataset). Thus, even while Osh7 displays cortical localization upon heat shock in vivo (Fig. 4e), it is possible that Osh proteins in addition to Osh3 are attenuated upon heat shock.

### PI4P metabolism controls localization of the exocyst component Exo70

Our results show that Osh proteins, including Osh3, control PI4P distribution at the PM. We therefore investigated roles for Stt4 and Osh3 in the polarized targeting of proteins at the PM. The exocyst subunit Exo70 localizes to sites of polarized growth including the bud tip and neck where it binds anionic lipids such as PI(4,5)P_2_ that is also enriched at sites of polarized growth [24, 25]. As PI4P metabolism impacts PI(4,5)P_2_ metabolism, alterations in PI4P localization may affect Exo70 localization at the PM. Consistent with this, Stt4 PI4K-generated PI4P is necessary for polarized targeting of Exo70 at the PM. Upon brief inactivation of the Stt4 PI4K (in *stt4* mutant cells at 32 °C for 10 min), Exo70-GFP is no longer exclusively found at sites of polarized growth (bud tips and necks). Instead, it is also observed at the PM in mother cells as well as more diffusely localized throughout the cytoplasm (Additional file 23: Figure S8a).

**Fig. 8:**
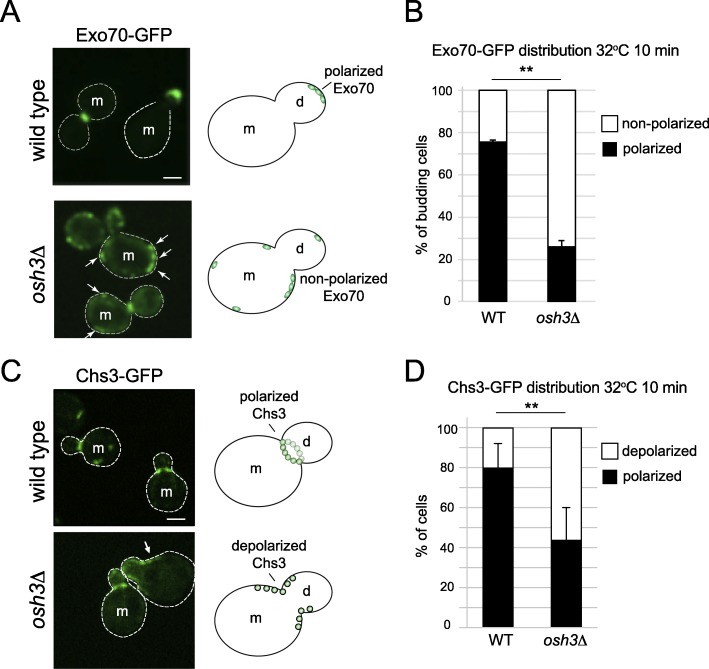
Osh3 regulates the polarized localization of the exocyst subunit Exo70 and polarized secretion of the chitin synthase Chs3. **a** Exponentially growing wild type or *osh3*∆ cells expressing Exo70-GFP from its endogenous promoter were incubated at 32 °C for 10 min just prior to being placed on a coverslip for imaging by spinning disk confocal microscopy. Representative confocal sections showing Exo70-GFP localization in wild type and *osh3*∆ mutant cells are provided. Arrows point to non-polarized Exo70-GFP foci in mother cells lacking Osh3 (*osh3*∆). Mother (m) and daughter (d) cells are indicated. Scale bar, 2 μm. **b** Quantitative analysis of Exo70-GFP polarization in small-budded cells after 10 min at 32 °C. Total number of cells analyzed: wild type 10 min 32 °C *n* = 182, *osh3*∆ 10 min 32 °C *n* = 146. The graph shows the means and standard deviations from three independent experiments (t test, **p < 0.002). **c** Exponentially growing wild type or *osh3*∆ cells expressing Chs3-GFP from its endogenous promoter were incubated at 32 °C for 10 min just prior to imaging by spinning disk confocal microscopy. Representative confocal sections showing Chs3-GFP localization in wild type and *osh3*∆ mutant cells are provided. Arrows point to cortical Chs3-GFP foci mislocalized from the mother-daughter neck in cells lacking Osh3 (*osh3*∆). Mother (m) and daughter (d) cells are indicated. Scale bar, 2 μm. **d** Quantitative analysis of Chs3-GFP mother-daughter neck localization in exponentially growing cells after 10 min at 32 °C. Total number of cells analyzed: wild type 10 min 32 °C *n* = 75, *osh3*∆ 10 min 32 °C *n* = 144. Graph shows the means and standard deviations from three independent experiments (t test, **p < 0.002)

To address whether Osh3 regulates Exo70 polarization, we examined Exo70 localization in wild type control cells and *osh3*∆ mutant cells. Heat shock is known to disrupt polarized secretion [15], and a brief heat shock at 42 °C results in depolarized Exo70 localization. Interestingly, cells lacking Osh3 displayed hypersensitivity to heat as assessed by monitoring Exo70-GFP localization. In control cells, Exo70-GFP was highly enriched at the tips of small-budded cells as well as bud necks in large-budded cells (Fig. 8a). Approximately 75% of control cells displayed polarized Exo70-GFP targeting at the tips of small-budded cells following a mild shift to 32 °C for 10 min (Fig. 8b and Additional file 24: Fig. 8b Dataset). In contrast, Exo70-GFP localization was not specified to bud tips in *osh3*∆ mutant cells (Fig. 8a). Less than 30% of the *osh3*∆ mutant cells displayed polarized targeting of Exo70-GFP to bud tips upon a mild shift to 32 °C (Fig. 8b and Additional file 24: Fig. 8b Dataset). Instead, cortical Exo70-GFP puncta were observed in > 70% of mother cells lacking Osh3. Moreover, the polarized targeting of Exo70-GFP to sites of polarized growth was impaired upon loss of Osh3 even at the non-stress growth temperature of 26 °C; only 50% of *osh3*∆ cells showed polarized Exo70 targeting versus 75% of control cells at 26 °C (Additional file 23: Figure S8b-c and Additional file 24: Fig. S8c Dataset).

Aberrant Exo70 polarization suggested that polarized secretion might be impaired upon loss of Osh3 function. To address this, we examined localization of the polarized secretory cargo protein Chs3-GFP in wild type control cells and *osh3*∆ mutant cells. Chs3 is a chitin synthase that undergoes cell cycle and exocyst-dependent delivery to bud tips and the bud neck region of the PM [42, 43]; heat stress results in depolarized Chs3 secretion [42]. Cells lacking Osh3 displayed hypersensitivity to heat as assessed by monitoring Chs3-GFP PM distribution. In wild type cells, Chs3-GFP PM targeting was specified to the bud tip and neck (Fig. 8c and Additional file 25: Fig. 8d Dataset). Among wild type cells displaying PM-localized Chs3-GFP, approximately 80% showed polarized Chs3-GFP targeting following a mild shift to 32 °C for 10 min (Fig. 8d and Additional file 25: Fig. 8d Dataset). In contrast, Chs3-GFP PM localization was no longer restricted to the bud neck in *osh3*∆ mutant cells (Fig. 8c). Among the mutant cells displaying PM-localized Chs3-GFP, less than 50% showed polarized Chs3-GFP targeting upon a mild shift to 32 °C (Fig. 8d and Additional file 25: Fig. 8d Dataset). Alternatively, Chs3-GFP mis-localization could be due to impaired endocytosis [43, 44], but *osh3*∆ mutant cells have not been reported to display strong endocytic defects [45]. Consistent with defects in polarized secretion, depolarized bud scars (chitin-rich sites of cell septation/division stained by calcofluor white) were also observed in mother cells lacking the Osh3 protein (Additional file 23: Figure S8d). Thus, the polarized distribution of PI4P, the exocyst component Exo70, the secretory cargo protein Chs3, and chitin deposits are altered in *osh3*∆ mutant cells. Altogether, these results suggest that PI4P metabolism controls the polarized targeting of secretory events at the PM and that cells lacking Osh3 are hyper-responsive to mild heat stress resulting in depolarized secretion.

## Discussion

### Phosphoinositide metabolism controls polarized cell growth

PI4P is enriched in the plasma membrane of rapidly growing small-budded yeast cells (during G1/S/G2) (Fig. 1). Likewise, PI(4,5)P_2_ is also enriched at sites of polarized growth in yeast (incipient bud sites, “shmoo” tips, and cleavage furrows) [24]. How PI4P and PI(4,5)P_2_ gradients are established is not well understood. Surprisingly, the Stt4 PI4K that synthesizes PI4P at the PM is found primarily in mother cells and is not readily apparent in small-budded daughter cells where PI4P is enriched [9, 10] (Figs. 1 and 2). This paradoxical difference in the distribution of the Stt4 PI4K and its product PI4P may be explained if PI4P pools generated in mother cells are rapidly turned over. Consistent with this notion, Stt4 PIK patches localize extensively to regions of the PM in proximity to the cortical ER containing the PI4P phosphatase Sac1 (Fig. 2 and Additional file 8: Figure S3).

Our data suggest that Stt4 distribution is mediated through interactions between the PIK patch component Efr3 and the ER-localized VAP orthologs Scs2/22. Loss of the Scs2/22 proteins does not disrupt Stt4 PIK patch formation at the PM, but does impair PIK patch ER association (Additional file 4: Figure S2). Accordingly, deletion of the FFAT motif in Efr3 reduced interaction with Scs2 (Fig. 3). To our knowledge, our study provides the first evidence that a PI4K, the yeast PI4KIIIα ortholog Stt4, resides at ER-PM contacts. It is unclear if mammalian PI4KIIIα localizes to ER-PM contacts, but phosphatidylinositol transfer proteins that regulate PI4KIIIα activity function at ER-PM contacts [46–48]. PI4P turnover also depends upon the ER-localized Scs2/22 proteins and the Sac1 phosphatase [16, 19, 49]. Thus, both PI4P synthesis and turnover may occur at ER-PM contacts. A recent study has suggested that Sac1 is not enriched at ER-PM contacts in HeLa cells [50]. However, the VAP isoforms are not thought to significantly contribute to ER-PM contact formation in HeLa cells [51]. Our findings and previous work indicate that (i) the Scs2/22 VAP orthologs are critical for cortical ER formation, (ii) the Sac1 phosphatase is present in the cortical ER, and (iii) Sac1 associates with Scs2 in the cortical ER in budding yeast cells (Additional file 8: Figure S3) [16, 32]. Thus, the findings from HeLa cells may not universally apply to ER-PM contacts in all cell types.

**Fig. 9:**
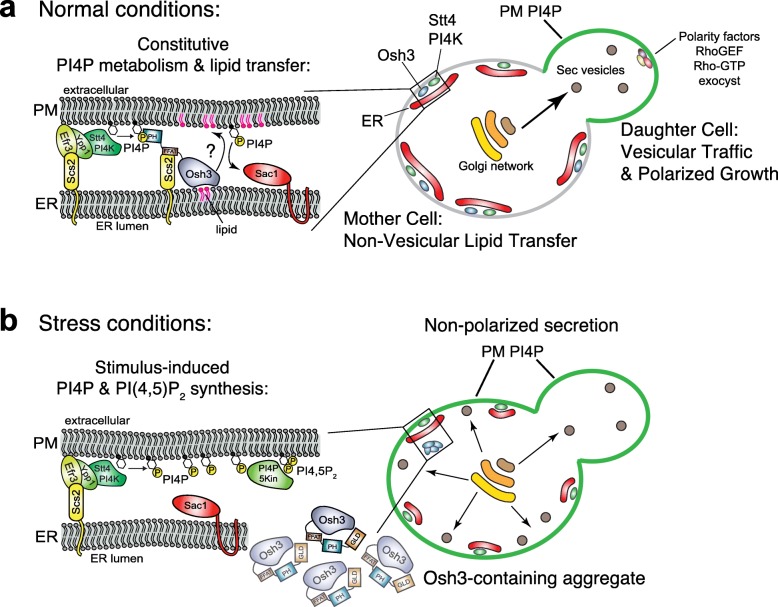
Speculative model for PI4P regulation during polarized growth versus stress conditions. **a** Under normal growth conditions, PM PI4P is regulated in mother cells at ER-PM contact sites through its production by Stt4 PIK patches and Osh protein-mediated turnover by Sac1. Osh3-mediated PI4P metabolism may also be coupled to exchange for an as yet unknown lipid at the PM and Osh3 function may also occur outside ER-PM contacts. PI4P levels at the PM are higher in the growing daughter cell than in the mother cell, possibly due to the lack of an established cortical ER network in the newly formed daughter cell. As a consequence, PI4P- and PI(4,5)P_2_-regulated vesicle trafficking is directed to the growing daughter cell. Plasma membrane maintenance in mother cells may be controlled by Osh protein-mediated non-vesicular lipid exchange [52]. **b** Under heat stress conditions, Osh3 localization and function at ER-PM contacts is impaired as Osh3 forms internal cytoplasmic aggregates. Diminished Osh3-mediated PI4P turnover contributes to increases in PI4P and possibly PI(4,5)P_2_ signals at the PM in mother cells. Loss of PI4P and PI(4,5)P_2_ polarization at the PM triggers isotropic (non-polarized) secretion, as well as additional stress responses necessary to maintain PM integrity

Placing PI4KIIIα at ER-PM junctions does not necessarily mean that PI4P synthesis and degradation occur in a futile cycle at ER-PM contacts. Rather, localization of the Stt4 PI4K at ER-PM contacts may provide insight into the regulation and function of PI4P at these cellular structures (Fig. 9a). The Scs2/22 VAP proteins bind and recruit the Osh2 and Osh3 proteins to ER-PM contacts [19, 35]. Osh2 and Osh3 are members of the ORP lipid exchange protein family proposed to deliver newly synthesized lipids from the ER to the PM in exchange for PI4P (see Fig. 2a). It remains to be established whether the Osh2 and Osh3 proteins transfer lipids between the ER and PM in vivo, but Osh3 can bind PI4P in vitro [36] and stimulate PI4P hydrolysis by the Sac1 phosphatase in vitro [19]. By positioning both PI 4-kinase and PI4P phosphatase activities at ER-PM contacts, the Osh2 and Osh3 proteins may be able to execute multiple rounds of non-vesicular lipid exchange reactions at a single site, and in doing so also control PI4P levels and distribution at the PM.

Importantly then, PI4P may serve at least two vital roles in membrane lipid dynamics: non-vesicular lipid exchange and vesicular trafficking. In mother cells with an extensive cortical ER network [31], PI4P may be consumed during Osh-mediated lipid exchange. Non-vesicular exchange of PI4P for another lipid does not result in a net lipid gain at the PM and may not directly drive rapid membrane expansion. However, PI4P exchange reactions may be critical for proper PM lipid composition and organization to ensure PM integrity in mother cells [17, 52], while polarized vesicular trafficking is directed to the growing bud (during G1/S/G2) [53]. Osh-mediated exchange reactions may also ensure that PI4P levels are kept low in mother cells, resulting in the polarized distribution of PI4P between mother and daughter cells. It is estimated that nearly 90% of Stt4-generated PI4P is rapidly consumed by the Sac1 phosphatase [49]; the bulk of this PI4P turnover may take place in mother cells via Osh-mediated reactions.

In contrast, PI4P is readily available at the PM of rapidly growing daughter cells. PI4P may serve as a spatial landmark along with PI(4,5)P_2_ and other anionic lipids for the targeting of polarity factors including Rho-family small GTPases, their associated guanine nucleotide exchange factors, and exocyst subunits that determine sites for the polarized targeting of secretory vesicles [9, 23, 25, 54]. Consistent with this idea, both PI4P and PI(4,5)P_2_ suffice for targeting polybasic proteins to the PM in mammalian cells [1]. While the ER is inherited in newly formed daughter cells, polarized trafficking of secretory vesicles continues and drives PM expansion in the growing bud until late G2/M, at which point the daughter cell establishes an extensive cortical ER network and switches from polarized to isotropic growth [31, 53]. The lack of an extensive cortical ER network (and the Sac1 phosphatase) may explain how PI4P accumulates in budding daughter cells that seem to be devoid of Stt4 PIK patches and even contain cortical Osh3 assemblies. Additional distinctions between mother and daughter cells may permit Osh-mediated PI4P regulation in mother cells but not daughters. In another speculative model, Osh3 may extract PI4P from the PM in mother cells or even from transport vesicles and then deposit PI4P at ER-free PM zones in daughter cells.

In simple terms, the PM may be considered as ER-associated and ER-free. Non-vesicular membrane lipid exchange may take place at ER-associated PM zones (predominately in mother cells), while vesicular membrane trafficking events occur in ER-free PM zones (i.e., polarized secretory trafficking to the growing bud) (Fig. 9a). ER-PM contacts are proposed to act as a physical barrier preventing exocytosis [55]. This is reasonable, as ER-PM contacts are closely apposed (10–30 nm apart) and may not accommodate secretory vesicles (50–100 nm in diameter). However, less than 50% of the PM is ER-associated in budding yeast and the cortical ER network is constantly reorganized [17]. In some mammalian tissue culture cells, including HeLa, only 2% of the PM is ER-associated [56] and it is unclear how the ER could serve as an effective fence to preclude vesicle docking and fusion at the PM.

We propose that control of PI4P metabolism and distribution by ORP/Osh family members may regulate, along with other factors, where sites of exocytosis occur. Upon heat shock, budding yeast cells rapidly halt polarized secretion to daughter cells and switch to isotropic trafficking between mother and daughter cells [15]. This coincides with a rapid increase in PI4P availability in mother cells (Fig. 1 and Additional file 1: Figure S1), possibly for heat-induced PI(4,5)P_2_ synthesis [9]. The Stt4 PI4K remains ER-associated upon heat shock (Additional file 11: Figure S4). Instead, Osh3 cortical localization decreases (Figs. 4 and 5), suggesting that attenuation of Osh3 contributes to generation of the PI4P signal in mother cells. In support of this, loss of Osh3 increases PI4P availability and Exo70 localization in mother cells (Figs. 4 and 8, Additional file 23: Figure S8).

Similar regulatory mechanisms may take place in mammalian cells. Depletion of ORP5 and ORP8 results in increased PI4P and PI(4,5)P_2_ levels [11, 14, 57, 58]. One recent study [58] suggests that ORP5/8 activity is tuned according to changes in PI(4,5)P_2_ levels. Upon PI(4,5)P_2_ depletion, for example by Ca^2+^-regulated phospholipase C activity [59, 60], ORP5 cortical localization is lost, likely due to reduced interactions between the ORP5 PH domain and PI(4,5)P_2_ at the PM [58]. Transient loss of ORP5/8 activity results in a rapid increase in PI4P for PI(4,5)P_2_ re-synthesis in order to maintain PI(4,5)P_2_ homeostasis. It will be interesting to examine whether mammalian ORP family members are attenuated to generate PI4P and PI(4,5)P_2_ signals in response to physiological stimuli that trigger regulated exocytosis.

### The Osh3 protein undergoes a heat-induced phase transition

Control of PI4P metabolism ensures cellular homeostasis and modulates responses to extracellular stimuli, such as a change in environmental conditions. We found that Osh3 undergoes a phase transition in response to heat shock. This may contribute to the rapid accumulation of PI4P at the PM under these conditions (Fig. 9b). Upon heat shock, Osh3 shifts from cortical assemblies to internal cytoplasmic aggregates containing the Hsp104 disaggregase (Fig. 5a). However, not all Hsp104 puncta contained Osh3, suggesting Osh3 may form a distinct subset of heat-sensitive aggregates (Fig. 5b). A growing number of proteins are known to undergo phase separations and transitions (e.g., liquid-liquid phase separation, reversible gel-like polymerization, and aggregation) in response to various stress conditions including heat stress and nutrient starvation. In budding yeast, these include the Whi3 protein upon prolonged pheromone exposure, the AMPK/Snf1 regulator Std1 in response to carbon source, and the Sup35 protein upon glucose starvation [61–63]. Another recent study found that the Pub1 protein exists in differential states in response to distinct stress conditions [40]. Pub1 forms reversible gel-like condensates upon a drop in pH and further converts to solid-state aggregates upon increasing temperature. Thus, it is increasingly clear that protein phase separations and protein aggregation have important physiological roles during cellular stress responses [64].

The Osh3 protein does not appear to contain hallmarks such as a prion-like domain or glutamine-rich region known to influence protein aggregation. However, it does possess differentially charged domains that may render it sensitive to changes in temperature and pH [40, 61]. The N-terminal GOLD and C-terminal ORD regions are quite basic (pI = 9.5 and 9.1, respectively) while the region spanning the FFAT motif is negatively charged (pI = 3.6). The PH and helical domains have pI values near physiological cytosolic pH under non-stress conditions (pI = 7.1 and 7.2, respectively). Importantly, cytosolic pH decreases upon heat shock as the PM H^+^-ATPase Pma1 is a heat-sensitive protein and the Pma1 inhibitor, Hsp30, is induced by heat [26, 65]. A reduction in cytosolic pH upon heat shock may induce Osh3 aggregation through inter-molecular electrostatic interactions between the negatively charged FFAT region and positively charged GOLD and ORD regions (the PH and HD regions would also become positively charged as cytoplasmic pH drops). Alternatively, proteins involved in multivalent interactions, like Osh3, may be prone to phase separations upon changes in environmental conditions [66]. As such, Osh3 aggregation may be promoted by interactions with other proteins that aggregate upon heat stress conditions. Curiously, our findings suggest that the Osh3 ORD region alone may form gel-like condensates in vitro upon increased temperature. However, the full-length Osh3 protein appears to form solid-state aggregates in vivo upon heat stress conditions. Because heat also induces a drop in cytosolic pH, full-length Osh3 may undergo a rapid conversion from gel-like condensates to solid-like stable aggregates, possibly explaining the differential in vitro and in vivo Osh3 material states. Potentially, Osh3 may undergo phase transitions and have different material properties (reversible gel-like polymers and stable solid-like aggregates) depending on different cellular and environmental conditions. It will be important to investigate whether ORP/Osh protein phase transitions modulate additional lipid signaling and membrane trafficking programs in response to various changes in physiological and environmental conditions.

## Conclusions

Our results suggest PI4P metabolism is a key determinant in the control of polarized secretion. Importantly, PI4P distribution can be rapidly modulated at the plasma membrane in response to changes in environmental conditions to direct polarized secretion as needed. Moreover, we find that PI4P polarization and availability may be regulated by a fascinating biophysical mechanism involving a heat-induced phase transition of a PI4P regulatory protein.

## Methods

### Yeast strains, plasmids, media, and growth assays

Descriptions of strains and plasmids used in this study are in Additional file 26: Table 1 and Additional file 27: Table 2. Gene deletions, truncations, and epitope tags were introduced into yeast by homologous recombination [67, 68]. The pRS vector series have been described previously [69]. Plasmids were sequenced to ensure that no mutations were introduced due to manipulations. Standard techniques and media were used for yeast and bacterial growth.

### Live yeast cell imaging

Fluorescence microscopy experiments were performed on mid-log yeast cultures in synthetic media at the indicated temperatures. Images for Fig. 2c, Additional file 4: Figure S2c, and Additional file 11: Figure S4a were obtained using a DeltaVision RT microscopy system (Applied Precision) equipped with an IX71 Olympus microscope, a PlanApo 100X objective (1.35 NA, Olympus), DAPI, FITC, and rhodamine filters, and a Cool Snap HQ digital camera (Photometrics). Images were deconvolved using soft-WoRx 3.5.0 software (Applied Precision, LLC). The brightness and contrast of images were linearly adjusted and cropped in Photoshop (Adobe) for presentation.

All other imaging data was acquired with a PerkinElmer Ultraview Vox spinning disk confocal microscope that consists of a Nikon TiE inverted stand attached to a Yokogawa CSU-X1 spinning disk scan head, a Hamamatsu C9100-13 EMCCD camera, Prior NanoscanZ piezo focus, and a Nikon Perfect Focus System (PFS). To measure PM PI4P intensities in the same cell before and after heat shock, cells expressing GFP-P4C were immobilized on agarose pads containing medium and 2% agarose. Cells were subsequently observed by microscopy during an in situ heat shock using a BIOPTECHS Objective Heater System (controller + heating collar). For lipid droplet visualization, 0.1 mM MDH (monodansyl pentane, Abgent) was added to mid-log cell cultures. After 15 min at room temperature, the cells were washed once with PBS. For staining chitin at bud scars, 0.05% (*w*:*v*) Calcofluor White (Fluorescent Brightener 28, Sigma) was added to mid-log cell cultures. After 15 min at room temperature, the cells were washed once with PBS. The numbers of cells observed in experiments are reported in the figures and figure legends. Original, unadjusted data was used for high-content quantitative analyses. The brightness and contrast of images were linearly adjusted and cropped in Photoshop (Adobe) for presentation.

### Quantitative image analysis

Quantitative image analysis was conducted using Fiji [70]. To measure PI4P intensities at the PM of mother and daughter cells (Fig. 1 and Additional file 1: Figure S1), cells expressing GFP-P4C and the PM marker mCherry-2xPH^PLCγ^ were analyzed at 26 °C and after a 10 min heat shock at 42 °C. Using Fiji, lines that passed through the cells were drawn and the corresponding fluorescence intensity profiles were plotted. The two highest GFP-P4C intensity values, which coincided with the mCherry-2xPH^PLCγ^ signal peaks in mother and daughter cells, were averaged and used to calculate Fd/Fm ratios for each cell.

To determine Stt4-ER association by high-content imaging (Fig. 2d), points of interests were identified using the Find Maxima tool applying appropriate noise tolerance settings in Fiji. The identified GFP signal maxima were selected and the intensity of the DsRed-HDEL (ER) signal for each GFP maxima was measured. To determine ER association of GFP maxima, the range of DsRed-HDEL signal intensities throughout the entire ER was identified using the threshold tool, defining a minimal threshold for the DsRed-HDEL (ER) signal. Likewise, maximal background DsRed-HDEL in ER-free regions was determined. Together, these measurements set a binary threshold to assign individual GFP maxima as either ER-associated or lacking ER.

Split GFP [71] signal intensity was measured using Fiji, utilizing co-expressed DsRed-HDEL (ER marker, Fig. 3a, b) or mCherry-2xPH PLC (PM marker, Fig. 3c, d) to select regions of interests. Osh3-GFP signal intensity (GFP fluorescence intensity) was measured using Fiji, utilizing co-expressed mCherry-2xPH ^PLCδ^ (PM marker) to select regions of interests. To quantify the co-localization of Osh3-GFP and Hsp104-mCherry at 26 °C and after a 10 min heat shock at 42 °C, the percent of pixel area with GFP fluorescence (above a set threshold) that overlapped with mCherry signal (above a set threshold) and vice versa was determined using Fiji.

### FRAP analyses

Photobleaching experiments were performed using an Ultraview Vox spinning disc confocal microscope with photokinesis unit and Volocity 6.3 software with FRAP module (Perkin Elmer, Seer Green, Beaconsfield, HP9 2FX). In vivo Osh3-GFP assemblies were bleached using the 488-nm laser at 100% intensity for 5 spot cycles with a spot period of 50 ms. In vitro NBD-labeled Osh3^588–996^ condensates were bleached for 5 spot cycles at 200 ms. The locations of bleach regions were defined using the Spot tool and saved as a FRAP template with the time-lapse data. The template was opened in Fiji [70], a threshold was applied to separate the bleach regions from the background, and converted to binary. The regions defined in the template were single pixel locations, but the laser bleached an area larger than a single pixel, so to reduce the effect of noise on the measurements the regions were dilated to 5 × 5 pixels before conversion into Fiji ROIs. The time-lapse data was opened in Fiji and corrected for camera bias and illumination inhomogeneity by subtracting a background image averaged over 100 frames to reduce noise. The ROIs were then applied, and the mean intensity time-lapse data measured for each region. Bleach region intensity values were corrected for incidental photobleaching during the time-lapse by normalizing to unbleached areas of fluorescence.

### Recombinant protein expression and purification

The bacterial expression vector pGEX6P-1 (GE healthcare) was used to express GST-Osh3^588–996^ and GST-Osh6 recombinant fusion proteins. The bacterial expression vector pRSETB was used to generate the his-Osh4 and his-Osh7 recombinant fusion proteins. *Escherichia coli* strains BL21 or Rosetta pLysS were used as a host cell line. Expression of recombinant protein was induced with 0.25 mM IPTG at 22 °C. The cell pellets were collected and resuspended in ice-cold homogenization buffer (50 mM Tris-HCl pH 6.8, 300 mM NaCl, 1 mM dithiothreitol (DTT), 0.1 mM AEBSF, and complete EDTA-free protease inhibitor). Cells were then disrupted by sonication in ice-cold homogenization buffer. The homogenized cells were centrifuged at 20,800×*g* for 30 min to remove cell debris. GST recombinant proteins were purified with glutathione-Sepharose. GST was cleaved from the Osh3^588–996^ and Osh6 proteins by using 0.1 U/μl PreScission protease. His-tagged recombinant proteins were purified with nickel-IMAC resin. Proteins were equilibrated with dialysis buffer (50 mM Tris-HCl pH 6.8, 150 mM NaCl, and 1 mM DTT) three times and then dialyzed with storage buffer (50 mM Tris-HCl pH 6.8, 150 mM NaCl, 2 mM DTT, and 50% glycerol) and stored at − 80 °C before analysis. Protein concentrations were determined by the Bradford assay (BioRad).

### Protein sedimentation assays

For the protein sedimentation assays (Additional file 21: Figure S7), purified Osh3^588–996^, his-Osh4, Osh6, his-Osh7, or NBD-labeled Osh3^588–996^ (see below) were incubated at the indicated temperature and pH for 10 min, and centrifuged at 50,000 rpm for 20 min at 25 °C. The resulting supernatant and pellet fractions were prepared for SDS-PAGE analysis and Coomassie-stained to detect recombinant proteins. The gels were scanned and relative amounts of fusion proteins in pellet (bound) and in supernatant (unbound) fractions were determined using Fiji [70].

### NBD-labeling of Osh3 ORD protein

Purified Osh3^588–996^ protein was dialyzed with 50 mM PBS pH 6.8, 150 mM NaCl, and 50% glycerol overnight before addition of IANBD amide (*N*,*N*′-dimethyl-*N*-(iodoacetyl)-*N*′-(7-nitrobenz-2-oxa-1,3-diazol-4-yl) ethylenediamine) (Thermo Fisher) at a final dye: protein ratio of 10:1 (mol:mol). The reaction was allowed to proceed for 2 h at room temperature and quenched by dialysis into 50 mM Tris-HCl pH 6.8, 150 mM NaCl, and 0.1% β-mercaptoethanol. NBD-labeled Osh3^588–996^ was stored at 4 °C before analysis.

### Fluorescence microscopy of purified Osh3 ORD aggregates

To generate the heat-induced condensates in Fig. 7, NBD-labeled Osh3^588–996^ was diluted to 10 μM in 50 mM Tris-HCl pH 6.8, 150 mM NaCl and incubated at either 26 °C or 42 °C for 15 min. In vitro assemblies were then immediately transferred to glass slides for imaging using the PerkinElmer Ultraview Vox spinning disk confocal microscope.

## Supplementary information

Additional file 1: Figure S1. PI4P distribution is regulated by growth conditions. (a) Cells expressing the PI4P reporter GFP-P4C (green) and a PM marker mCherry-2xPH^PLCδ^ (magenta) were grown at 26 °C (left panels) and subjected to a heat shock for 10 min at 42 °C (right panels). Mother cells are indicated (m) and arrows point to GFP-P4C localization at the PM of mother cells at 42 °C. Scale bars, 5 μm. (b) Representative images of a time course of cells expressing the PI4P reporter GFP-P4C subjected to a heat shock at 42 °C. Cells were grown at 26 °C, immobilized on a 2% agarose pad mounted on a microscope slide. Arrowheads point to GFP-P4C localization at the PM of mother cells. Cells were imaged over time at 42 °C using a BIOPTECHS Objective Heater System. Scale bar, 5 μm. (c) Graph displays the mean Fd/Fm ratios of GFP-P4C fluorescence at 26 °C (t=0) and during heat shock at 42 °C for different time points (2 min intervals, see B). Error bars represent standard deviation. In total, 10 cells from two independent experiments were analyzed. (d) Graph shows the mean GFP-P4C fluorescence intensity at the mother cell PM (Fm) at 26 °C (t=0) and during heat shock at 42 °C at different time points (2 min intervals, see b and c). In total, 10 cells from two independent experiments were analyzed.

Additional file 2: Fig. 1c and 1d Dataset

Additional file 3: Fig. S1c and S1d Dataset

Additional file 4: Figure S2. Stt4 PIK patches localize to ER-PM contact sites and contribute to heat stress-induced PI4P signaling. (a) The Stt4 PI4K generates PI4P at the PM. Wild type cells (upper panel) and temperature conditional *stt4-4* cells (lower panel) expressing the PI4P reporter GFP-P4C grown at 26 °C and after heat shock at 42 °C. Arrows point to GFP-P4C localization at the PM of mother cells at 42 °C. Scale bars, 5 μm. (b) Schematic representation of the method used to measure PM GFP-P4C fluorescence intensities at 34 °C and after 42 °C heat shock (left). Briefly, line scans were applied through both daughter and mother cells using Fiji and the peak values corresponding to the GFP-P4C fluorescence intensity at the PM in the daughter (Fd) and mother cell (Fm) were recorded to calculate Fd/Fm ratios. Graph shows the Fd/Fm ratio of individual cells at 34 °C and after a 10 min heat shock at 42 °C. Total number of cells analyzed: wild type 34 °C *n*=97, wild type 10min 42 °C *n*=160, *ypp1-7* 34 °C *n*=118, *ypp1-7* 10min 42 °C *n*=123. Mean values and standard deviations from four independent experiments are shown (one-way ANOVA, ****p<0.0001). (c) Localization of GFP-Stt4 (green) and the ER marker DsRed-HDEL (magenta) in *rtn1*∆ *rtn2*∆ *yop1*∆ mutant cells (upper panel) or *scs2*∆ *scs22*∆ (lower panel) mutant cells. In *rtn1*∆ *rtn2*∆ *yop1*∆ mutant cells, Stt4 PIK patches (outlined in white) are associated with the cortical ER (magenta) and are absent from ER-free PM zones (dashed lines). In *scs2*∆ *scs22*∆ mutant cells, Stt4 PIK patches (marked by asterisks) are found in ER-free PM zones (dashed lines). The arrow points to a PIK patch associated with the cortical ER (merge) in *scs2*∆ *scs22*∆ mutant cells. Thus, Stt4 may also localize to Scs2/22-independent ER-PM contacts, consistent with distinct Stt4 complexes (I and II; see Figure 2a). Scale bar, 4 μm.

Additional file 5: Fig. S2b Dataset

Additional file 6: Fig. 2c and S2c Dataset

Additional file 7: Fig. 2d Dataset

Additional file 8: Figure S3. ER-localized Scs2 interacts with the PIK patch subunit Efr3 at the PM and the PI4P phosphatase Sac1 in the ER. (a) Cartoon displaying the principle of BiFC using the split GFP assay. The N-terminal half of GFP (GFP_N_) and the C-terminal half of GFP (GFP_C_) only form a fluorescent GFP when brought into spatial proximity if their fusion partners, protein A and protein B, interact with each other. (b) Protein-protein interactions between Efr3, Scs2, Sac1, and Ypp1 as detected by the split GFP BiFC assay. In each case, GFP_N_ is fused to the protein on listed on the left and GFP_C_ is fused to the protein on listed on the right. In the Efr3-Sac1 pairing, for example, cells express Efr3-GFP_N_ and Sac1-GFP_C_. The pseudo-colored images (intensity maps) indicate the scale of specific interactions (blue, moderate; red, strong). Scale bars, 3 μm

Additional file 9: Fig. 3b Dataset

Additional file 10: Fig. 3d Dataset

Additional file 11: Figure S4. Heat shock does not disrupt the cortical ER network, Stt4 localization at ER-PM contacts, or the cortical localization of Osh2 and Osh7. (a) Wild type cells expressing the PI4P reporter GFP-2xPH^Osh2^ (green) and the ER marker DsRed-HDEL (magenta) were grown at 26 °C (left panels) and subjected to a heat shock at 42 °C for 10 minutes (right panels). Cortical ER is present and observed under both conditions. (b) Top view images of a cell expressing GFP-Stt4 (green) and the ER marker DsRed-HDEL (magenta) after a heat shock for 10 min at 42 °C. (c) Representative examples of wild type, *osh6*∆, *osh7*∆, and *osh2*∆ cells expressing the PI4P reporter GFP-P4C grown at 26 °C to mid-log phase. GFP-P4C fluorescence intensities at the plasma membrane of daughter cells (Fd) and mother cells were measured as indicated and corresponding Fd/Fm ratios for the cells shown are indicated under each image. The periphery of the wild type mother cell is indicated (dashed white line). Arrows point to the PI4P reporter at the PM in mother cells. Scale bar, 2 μm.

Additional file 12: Fig. 4b Dataset

Additional file 13: Fig. 4d Dataset

Additional file 14: Fig. 5b Dataset

Additional file 15: Figure S5. Heat stress-induced Osh3-GFP aggregates do not co-localize with membrane-bound organelles. (a) Cells expressing Osh3-GFP (green) under its endogenous promoter were co-labelled with established markers of various different organelles (magenta): mRFP-Sed5 (early Golgi compartments), mRFP-Gos1 (medial Golgi compartments), Sec7-DsRed (late Golgi compartments), mRFP-FYVE (PI3P-containing endosomes), DsRed-HDEL (endoplasmic reticulum; ER) and MDH (lipid droplets). Cells were grown at 26 °C and then shifted 10 min at 42 °C prior to imaging. Scale bar, 2 μm. (b) Wild type cells expressing GFP were grown at 26 °C (left panel) and subjected to a heat shock for 10 min at 42 °C (right panel). Scale bar, 3 μm. (PDF 638 kb)

Additional file 16: Fig. 6a Dataset

Additional file 17: Fig. 6c Dataset

Additional file 18: Osh3 Localization Dataset

Additional file 19: Figure S6. The Osh3 GOLD and ORD regions aggregate upon brief heat stress conditions. (a) Schematic representations and cellular localization of full length Osh3-GFP and the C-terminal Osh3 truncation protein GOLD-GFP. The truncation was performed by homologous recombination and both proteins were expressed from the *OSH3* promoter. Abbreviations shown are: GOLD, Golgi dynamics domain; PH, pleckstrin homology domain; HD, helical domain; FFAT, two phenyalanines in an acidic tract; ORD, OSBP-related domain; GFP, green fluorescent protein. Cells expressing full length Osh3-GFP or GOLD-GFP were grown at 26 °C and then shifted to 37 °C or 42 °C for 10 min prior to imaging by spinning disk confocal microscopy. Scale bar, 2 μm. (b) Schematic representations and cellular localization of full length Osh3-GFP and the N-terminal Osh3 truncation protein ORD-GFP. The truncation was performed by homologous recombination and both proteins were expressed from the *ADH1* promoter. Abbreviations are the same as in Figure S6a. Cells expressing full length Osh3-GFP or ORD-GFP were grown at 26 °C and then shifted to 37 °C or 42 °C for 10 min prior to imaging by spinning disk confocal microscopy. Scale bar, 2 μm. (c) Localization of the PI4P reporter mCherry-P4C FLARE (magenta) in cells expressing either full length Osh3-GFP (green) or a truncated Osh3 protein lacking the ORD domain (GOLD-PH-HD-FFAT-GFP, green). The truncation was performed by homologous recombination and both proteins were expressed from the *OSH3* promoter. Corresponding Fd/Fm ratios for the cells shown are indicated in each image. Arrow points to PI4P at the PM in a mother cell. Abbreviations are the same as in Figure S6a. Cells were grown at 26 °C to mid-log phase prior to imaging by spinning disk confocal microscopy. Scale bar, 2 μm.

Additional file 20: Fig. 7c Dataset

Additional file 21: Figure S7. The PI4P-binding ORD region of Osh proteins is heat sensitive in vitro. (a) (Top panel) Schematic representations of full length Osh3, Osh4, Osh6 and Osh7. Abbreviations: GOLD, Golgi dynamics domain; PH, pleckstrin homology domain; HD, helical domain; FFAT, two phenyalanines in an acidic tract; ORD, OSBP-related domain. (Bottom panels) The ORD region of Osh proteins sediments at elevated temperature. Purified Osh3^588–996^, his-Osh4, Osh6 and his-Osh7 were subjected to incubation at the indicated temperatures for 10 min prior to ultracentrifugation. P, pellet fraction; S, supernatant fraction. Quantitations of fractions are the averages and standard deviations from three independent experiments. (b) NBD-labelled Osh3^588-996^ sediments at elevated temperature. Purified NBD-labelled Osh3^588-996^ (see Figure 7) was subjected to incubation at the indicated temperatures for 10min prior to ultracentrifugation. P, pellet fraction; S, supernatant fraction.

Additional file 22: Fig. S7a Dataset

Additional file 23: Figure S8. Osh3 regulates the polarized localization of the exocyst subunit Exo70 and polarized secretion of the chitin synthase Chs3. (a) Wild type and temperature conditional *stt4-4* mutant cells expressing Exo70-GFP at 26 °C were grown to log phase at 26 °C, shifted 10 min at 32 °C, and then imaged by spinning disk confocal microscopy. Representative confocal sections showing Exo70-GFP localization in wild type and *stt4-4* mutant cells and corresponding Nomarski images are provided. Arrows point to non-polarized Exo70-GFP foci in *stt4-4* mother cells. Mother (m) and daughter (d) cells are indicated. Scale bar, 2 μm. (b) Exponentially growing wild type or *osh3*∆ cells expressing Exo70-GFP at 26 °C were imaged by spinning disk confocal microscopy. Representative confocal sections showing Exo70-GFP localization in wild type and *osh3*∆ mutant cells are provided. Arrows point to non-polarized Exo70-GFP foci in mother cells lacking Osh3 (*osh3*∆). Mother (m) and daughter (d) cells are indicated. Scale bar, 2 μm. (c) Quantitative analysis of Exo70-GFP polarization in small-budded cells at 26 °C. Total number of cells analyzed: wild type 26 °C *n*=212, *osh3*∆ 26 °C *n*=208. The graph shows the means and standard deviations from three independent experiments (t test, *p< 0.015). (d) Exponentially growing wild type and *osh3*∆ cells were stained with calcofluor white (a dye that stains cell wall chitin enriched at sites of cell division known as bud scars). Representative Z projections showing bud scar distribution are provided. Arrow shows a non-polarized bud scar in an *osh3*∆ mutant cell. Scale bar, 2 μm.

Additional file 24: Fig. 8b and S8c Dataset

Additional file 25: Fig. 8d Dataset

Additional file 26: Table 1

Additional file 27: Table 2

## Abbreviations

ER-PM contacts: Endoplasmic reticulum-plasma membrane contacts
GOLD domain: Golgi dynamics domain
ORD: Oxysterol-binding protein-related domain
Osh protein: Oxysterol-binding protein homology protein
PI4P: Phosphatidylinositol 4-phosphate

## References

[1] Hammond GR, Fischer MJ, Anderson KE, Holdich J, Koteci A, Balla T, Irvine RF (2012). PI4P and PI(4,5)P_2_ are essential but independent lipid determinants of membrane identity. Science.

[2] Chavez M, Ena S, Van Sande J, de Kerchove d'Exaerde A, Schurmans S, Schiffmann SN (2015). Modulation of ciliary phosphoinositide content regulates trafficking and sonic hedgehog signaling output. Dev Cell.

[3] Garcia-Gonzalo FR, Phua SC, Roberson EC, Garcia G, Abedin M, Schurmans S, Inoue T, Reiter JF (2015). Phosphoinositides regulate ciliary protein trafficking to modulate hedgehog signaling. Dev Cell.

[4] Ghugtyal V, Garcia-Rodas R, Seminara A, Schaub S, Bassilana M, Arkowitz RA (2015). Phosphatidylinositol-4-phosphate-dependent membrane traffic is critical for fungal filamentous growth. Proc Natl Acad Sci U S A.

[5] Audhya A, Foti M, Emr SD (2000). Distinct roles for the yeast phosphatidylinositol 4-kinases, Stt4p and Pik1p, in secretion, cell growth, and organelle membrane dynamics. Mol Biol Cell.

[6] Wild AC, Yu JW, Lemmon MA, Blumer KJ (2004). The p21-activated protein kinase-related kinase Cla4 is a coincidence detector of signaling by Cdc42 and phosphatidylinositol 4-phosphate. J Biol Chem.

[7] Balla A, Tuymetova G, Tsiomenko A, Varnai P, Balla T (2005). A plasma membrane pool of phosphatidylinositol 4-phosphate is generated by phosphatidylinositol 4-kinase type-III alpha: studies with the PH domains of the oxysterol binding protein and FAPP1. Mol Biol Cell.

[8] Nakatsu F, Baskin JM, Chung J, Tanner LB, Shui G, Lee SY, Pirruccello M, Hao M, Ingolia NT, Wenk MR, De Camilli P (2012). PtdIns4P synthesis by PI4KIIIalpha at the plasma membrane and its impact on plasma membrane identity. J Cell Biol.

[9] Audhya A, Emr SD (2002). Stt4 PI 4-kinase localizes to the plasma membrane and functions in the Pkc1-mediated MAP kinase cascade. Dev Cell.

[10] Baird D, Stefan C, Audhya A, Weys S, Emr SD (2008). Assembly of the PtdIns 4-kinase Stt4 complex at the plasma membrane requires Ypp1 and Efr3. J Cell Biol.

[11] Chung J, Torta F, Masai K, Lucast L, Czapla H, Tanner LB, Narayanaswamy P, Wenk MR, Nakatsu F, De Camilli P (2015). INTRACELLULAR TRANSPORT. PI4P/phosphatidylserine countertransport at ORP5- and ORP8-mediated ER-plasma membrane contacts. Science.

[12] Moser von Filseck J, Copic A, Delfosse V, Vanni S, Jackson CL, Bourguet W, Drin G (2015). INTRACELLULAR TRANSPORT. Phosphatidylserine transport by ORP/Osh proteins is driven by phosphatidylinositol 4-phosphate. Science.

[13] Schulz TA, Choi MG, Raychaudhuri S, Mears JA, Ghirlando R, Hinshaw JE, Prinz WA (2009). Lipid-regulated sterol transfer between closely apposed membranes by oxysterol-binding protein homologues. J Cell Biol.

[14] Sohn M, Ivanova P, Brown HA, Toth DJ, Varnai P, Kim YJ, Balla T (2016). Lenz-Majewski mutations in PTDSS1 affect phosphatidylinositol 4-phosphate metabolism at ER-PM and ER-Golgi junctions. Proc Natl Acad Sci U S A.

[15] Delley PA, Hall MN (1999). Cell wall stress depolarizes cell growth via hyperactivation of RHO1. J Cell Biol.

[16] Manford AG, Stefan CJ, Yuan HL, Macgurn JA, Emr SD (2012). ER-to-plasma membrane tethering proteins regulate cell signaling and ER morphology. Dev Cell.

[17] Omnus DJ, Manford AG, Bader JM, Emr SD, Stefan CJ (2016). Phosphoinositide kinase signaling controls ER-PM cross-talk. Mol Biol Cell.

[18] Sorensen DM, Holen HW, Pedersen JT, Martens HJ, Silvestro D, Stanchev LD, Costa SR, Gunther Pomorski T, Lopez-Marques RL, Palmgren M (2019). The P5A ATPase Spf1p is stimulated by phosphatidylinositol 4-phosphate and influences cellular sterol homeostasis. Mol Biol Cell.

[19] Stefan CJ, Manford AG, Baird D, Yamada-Hanff J, Mao Y, Emr SD (2011). Osh proteins regulate phosphoinositide metabolism at ER-plasma membrane contact sites. Cell.

[20] Roy A, Levine TP (2004). Multiple pools of phosphatidylinositol 4-phosphate detected using the pleckstrin homology domain of Osh2p. J Biol Chem.

[21] Luo X, Wasilko DJ, Liu Y, Sun J, Wu X, Luo ZQ, Mao Y (2015). Structure of the Legionella virulence factor, SidC reveals a unique PI (4) P-specific binding domain essential for its targeting to the bacterial Phagosome. PLoS Pathog.

[22] Wills RC, Goulden BD, Hammond GRV (2018). Genetically encoded lipid biosensors. Mol Biol Cell.

[23] Fairn GD, Hermansson M, Somerharju P, Grinstein S (2011). Phosphatidylserine is polarized and required for proper Cdc42 localization and for development of cell polarity. Nat Cell Biol.

[24] Garrenton LS, Stefan CJ, McMurray MA, Emr SD, Thorner J (2010). Pheromone-induced anisotropy in yeast plasma membrane phosphatidylinositol-4,5-bisphosphate distribution is required for MAPK signaling. Proc Natl Acad Sci U S A.

[25] He B, Xi F, Zhang X, Zhang J, Guo W (2007). Exo70 interacts with phospholipids and mediates the targeting of the exocyst to the plasma membrane. EMBO J.

[26] Zhao Y, Macgurn JA, Liu M, Emr S (2013). The ART-Rsp5 ubiquitin ligase network comprises a plasma membrane quality control system that protects yeast cells from proteotoxic stress. Elife.

[27] Baskin JM, Wu X, Christiano R, Oh MS, Schauder CM, Gazzerro E, Messa M, Baldassari S, Assereto S, Biancheri R, Zara F, Minetti C, Raimondi A, Simons M, Walther TC, Reinisch KM, De Camilli P (2016). The leukodystrophy protein FAM126A (hyccin) regulates PtdIns (4) P synthesis at the plasma membrane. Nat Cell Biol.

[28] Chung J, Nakatsu F, Baskin JM, De Camilli P (2015). Plasticity of PI4KIIIalpha interactions at the plasma membrane. EMBO Rep.

[29] Zhai C, Li K, Markaki V, Phelan JP, Bowers K, Cooke FT, Panaretou B (2008). Ypp1/YGR198w plays an essential role in phosphoinositide signalling at the plasma membrane. Biochem J.

[30] Balakrishnan SS, Basu U, Shinde D, Thakur R, Jaiswal M, Raghu P. Regulation of PI4P levels by PI4KIIIalpha during G-protein coupled PLC signaling in Drosophila photoreceptors. J Cell Sci. 2018;131 10.1242/jcs217257. 10.1242/jcs.217257 PMC6104824 29980590

[31] West M, Zurek N, Hoenger A, Voeltz GK (2011). A 3D analysis of yeast ER structure reveals how ER domains are organized by membrane curvature. J Cell Biol.

[32] Loewen CJ, Young BP, Tavassoli S, Levine TP (2007). Inheritance of cortical ER in yeast is required for normal septin organization. J Cell Biol.

[33] Murphy SE, Levine TP (2016). VAP, a Versatile Access Point for the Endoplasmic Reticulum: Review and analysis of FFAT-like motifs in the VAPome. Biochim Biophys Acta.

[34] Wu X, Chi RJ, Baskin JM, Lucast L, Burd CG, De Camilli P, Reinisch KM (2014). Structural insights into assembly and regulation of the plasma membrane phosphatidylinositol 4-kinase complex. Dev Cell.

[35] Loewen CJ, Levine TP (2005). A highly conserved binding site in vesicle-associated membrane protein-associated protein (VAP) for the FFAT motif of lipid-binding proteins. J Biol Chem.

[36] Tong J, Yang H, Eom SH, Im YJ (2013). Structure of Osh3 reveals a conserved mode of phosphoinositide binding in oxysterol-binding proteins. Structure.

[37] Sweeny EA, Shorter J (2016). Mechanistic and structural insights into the prion-disaggregase activity of Hsp104. J Mol Biol.

[38] Alberti S, Gladfelter A, Mittag T (2019). Considerations and challenges in studying liquid-liquid phase separation and biomolecular condensates. Cell.

[39] Alberti S, Saha S, Woodruff JB, Franzmann TM, Wang J, Hyman AA (2018). A User's guide for phase separation assays with purified proteins. J Mol Biol.

[40] Kroschwald S, Munder MC, Maharana S, Franzmann TM, Richter D, Ruer M, Hyman AA, Alberti S (2018). Different material states of Pub1 condensates define distinct modes of stress adaptation and recovery. Cell Rep.

[41] Escusa-Toret S, Vonk WI, Frydman J (2013). Spatial sequestration of misfolded proteins by a dynamic chaperone pathway enhances cellular fitness during stress. Nat Cell Biol.

[42] Valdivia RH, Schekman R (2003). The yeasts Rho1p and Pkc1p regulate the transport of chitin synthase III (Chs3p) from internal stores to the plasma membrane. Proc Natl Acad Sci U S A.

[43] Zanolari B, Rockenbauch U, Trautwein M, Clay L, Barral Y, Spang A (2011). Transport to the plasma membrane is regulated differently early and late in the cell cycle in Saccharomyces cerevisiae. J Cell Sci.

[44] Reyes A, Sanz M, Duran A, Roncero C (2007). Chitin synthase III requires Chs4p-dependent translocation of Chs3p into the plasma membrane. J Cell Sci.

[45] Encinar Del Dedo J, Idrissi FZ, Fernandez-Golbano IM, Garcia P, Rebollo E, Krzyzanowski MK, Grotsch H, Geli MI (2017). ORP-mediated ER contact with endocytic sites facilitates actin polymerization. Dev Cell.

[46] Chang CL, Hsieh TS, Yang TT, Rothberg KG, Azizoglu DB, Volk E, Liao JC, Liou J (2013). Feedback regulation of receptor-induced Ca2+ signaling mediated by E-Syt1 and Nir2 at endoplasmic reticulum-plasma membrane junctions. Cell Rep.

[47] Kim YJ, Guzman-Hernandez ML, Wisniewski E, Balla T (2015). Phosphatidylinositol-phosphatidic acid exchange by Nir2 at ER-PM contact sites maintains phosphoinositide signaling competence. Dev Cell.

[48] Lees JA, Messa M, Sun EW, Wheeler H, Torta F, Wenk MR, De Camilli P, Reinisch KM. Lipid transport by TMEM24 at ER-plasma membrane contacts regulates pulsatile insulin secretion. Science. 2017;355(6326):eaah6171. 10.1126/science.aah6171 PMC5414417 28209843

[49] Foti M, Audhya A, Emr SD (2001). Sac1 lipid phosphatase and Stt4 phosphatidylinositol 4-kinase regulate a pool of phosphatidylinositol 4-phosphate that functions in the control of the actin cytoskeleton and vacuole morphology. Mol Biol Cell.

[50] Zewe JP, Wills RC, Sangappa S, Goulden BD, Hammond GR (2018). SAC1 degrades its lipid substrate PtdIns4P in the endoplasmic reticulum to maintain a steep chemical gradient with donor membranes. Elife.

[51] Dong R, Saheki Y, Swarup S, Lucast L, Harper JW, De Camilli P (2016). Endosome-ER contacts control actin nucleation and Retromer function through VAP-dependent regulation of PI4P. Cell.

[52] Nishimura T, Gecht M, Covino R, Hummer G, Surma MA, Klose C, Arai H, Kono N, Stefan CJ. Osh proteins control nanoscale lipid organization necessary for PI(4,5)P_2_ synthesis. Mol Cell. 2019;75(5):1043–57. e8. 10.1016/j.molcel.2019.06.037 PMC6739424 31402097

[53] McCusker D, Kellogg DR (2012). Plasma membrane growth during the cell cycle: unsolved mysteries and recent progress. Curr Opin Cell Biol.

[54] Zhang X, Orlando K, He B, Xi F, Zhang J, Zajac A, Guo W (2008). Membrane association and functional regulation of Sec3 by phospholipids and Cdc42. J Cell Biol.

[55] Ng AYE, Ng AQE, Zhang D (2018). ER-PM contacts restrict exocytic sites for polarized morphogenesis. Curr Biol.

[56] Giordano F, Saheki Y, Idevall-Hagren O, Colombo SF, Pirruccello M, Milosevic I, Gracheva EO, Bagriantsev SN, Borgese N, De Camilli P. PI(4,5)P_2_-dependent and Ca (2+)-regulated ER-PM interactions mediated by the extended synaptotagmins. Cell. 2013;153(7):1494–509. 10.1016/j.cell.2013.05.026 PMC3716012 23791178

[57] Ghai R, Du X, Wang H, Dong J, Ferguson C, Brown AJ, Parton RG, Wu JW, Yang H (2017). ORP5 and ORP8 bind phosphatidylinositol-4, 5-biphosphate (PtdIns (4,5) P 2) and regulate its level at the plasma membrane. Nat Commun.

[58] Sohn M, Korzeniowski M, Zewe JP, Wills RC, Hammond GRV, Humpolickova J, Vrzal L, Chalupska D, Veverka V, Fairn GD, Boura E, Balla T. PI(4,5)P_2_ controls plasma membrane PI4P and PS levels via ORP5/8 recruitment to ER-PM contact sites. J Cell Biol. 2018;217(5):1797–813. 10.1083/jcb.201710095 PMC5940310 29472386

[59] Toth JT, Gulyas G, Toth DJ, Balla A, Hammond GR, Hunyady L, Balla T, Varnai P (2016). BRET-monitoring of the dynamic changes of inositol lipid pools in living cells reveals a PKC-dependent PtdIns4P increase upon EGF and M3 receptor activation. Biochim Biophys Acta.

[60] Varnai P, Balla T (1998). Visualization of phosphoinositides that bind pleckstrin homology domains: calcium- and agonist-induced dynamic changes and relationship to myo-[3H]inositol-labeled phosphoinositide pools. J Cell Biol.

[61] Franzmann TM, Jahnel M, Pozniakovsky A, Mahamid J, Holehouse AS, Nuske E, Richter D, Baumeister W, Grill SW, Pappu RV, Hyman AA, Alberti S (2018). Phase separation of a yeast prion protein promotes cellular fitness. Science.

[62] Simpson-Lavy K, Xu T, Johnston M, Kupiec M (2017). The Std1 activator of the Snf1/AMPK kinase controls glucose response in yeast by a regulated protein aggregation. Mol Cell.

[63] Schlissel G, Krzyzanowski MK, Caudron F, Barral Y, Rine J (2017). Aggregation of the Whi3 protein, not loss of heterochromatin, causes sterility in old yeast cells. Science.

[64] Franzmann TM, Alberti S. Protein phase separation as a stress survival strategy. Cold Spring Harb Perspect Biol. 2019;11(6):a034058. 10.1101/cshperspect.a034058 PMC6546044 30617047

[65] Panaretou B, Piper PW (1992). The plasma membrane of yeast acquires a novel heat-shock protein (hsp30) and displays a decline in proton-pumping ATPase levels in response to both heat shock and the entry to stationary phase. Eur J Biochem.

[66] Wang J, Choi JM, Holehouse AS, Lee HO, Zhang X, Jahnel M, Maharana S, Lemaitre R, Pozniakovsky A, Drechsel D, Poser I, Pappu RV, Alberti S, Hyman AA (2018). A molecular grammar governing the driving forces for phase separation of prion-like RNA binding proteins. Cell.

[67] Janke C, Magiera MM, Rathfelder N, Taxis C, Reber S, Maekawa H, Moreno-Borchart A, Doenges G, Schwob E, Schiebel E, Knop M (2004). A versatile toolbox for PCR-based tagging of yeast genes: new fluorescent proteins, more markers and promoter substitution cassettes. Yeast.

[68] Longtine MS, McKenzie A, Demarini DJ, Shah NG, Wach A, Brachat A, Philippsen P, Pringle JR (1998). Additional modules for versatile and economical PCR-based gene deletion and modification in Saccharomyces cerevisiae. Yeast.

[69] Sikorski RS, Hieter P (1989). A system of shuttle vectors and yeast host strains designed for efficient manipulation of DNA in Saccharomyces cerevisiae. Genetics.

[70] Schindelin J, Arganda-Carreras I, Frise E, Kaynig V, Longair M, Pietzsch T, Preibisch S, Rueden C, Saalfeld S, Schmid B, Tinevez JY, White DJ, Hartenstein V, Eliceiri K, Tomancak P, Cardona A (2012). Fiji: an open-source platform for biological-image analysis. Nat Methods.

[71] Barnard E, McFerran NV, Trudgett A, Nelson J, Timson DJ (2008). Detection and localisation of protein-protein interactions in Saccharomyces cerevisiae using a split-GFP method. Fungal Genet Biol.

