# Supplementary figures and images for: A heat-sensitive Osh protein controls PI4P polarity
Source: BMC Biol. 2020 Mar 13;18:28. doi: 10.1186/s12915-020-0758-x (PMC7071650; doi:10.1186/s12915-020-0758-x)

**a**

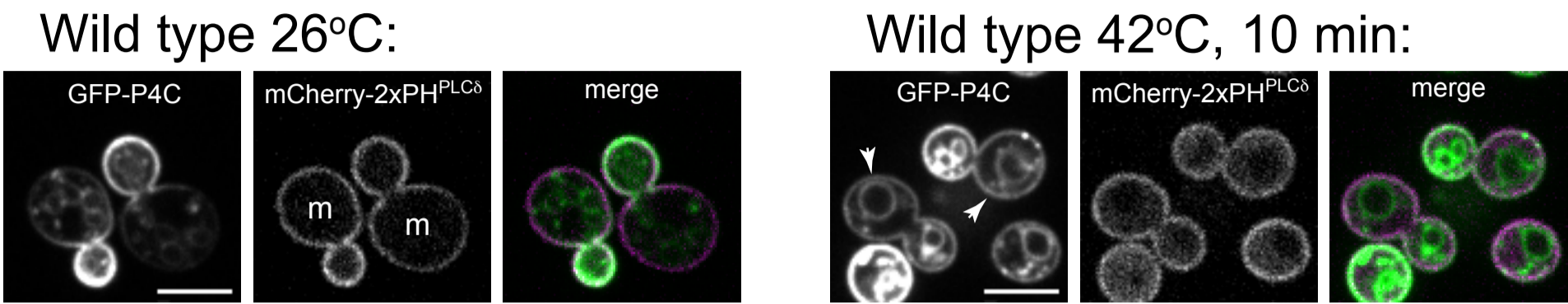

**b**

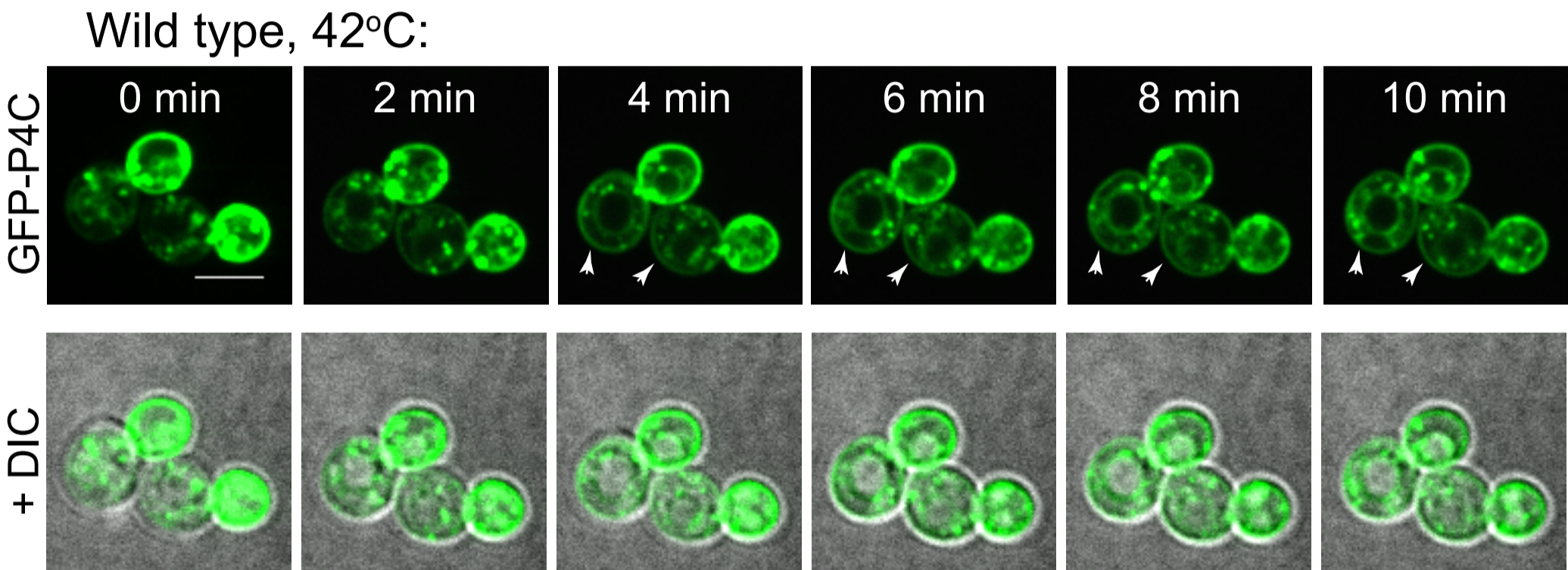

**c**

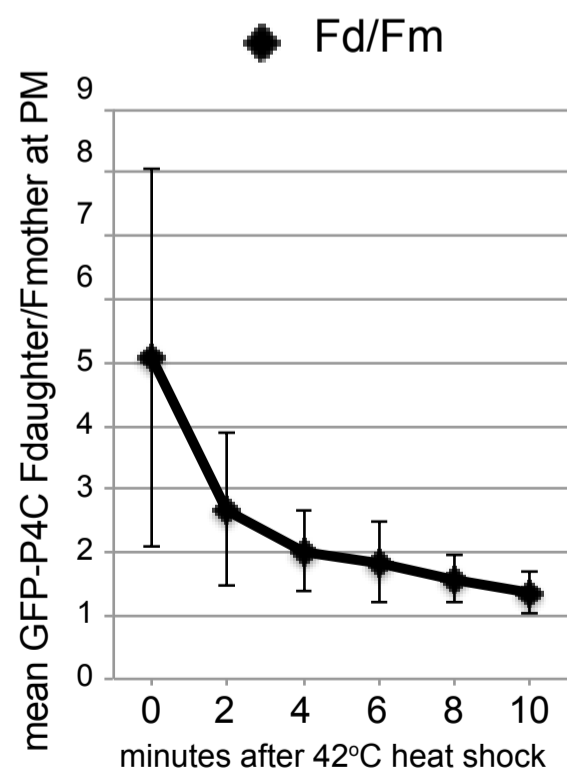

**d**

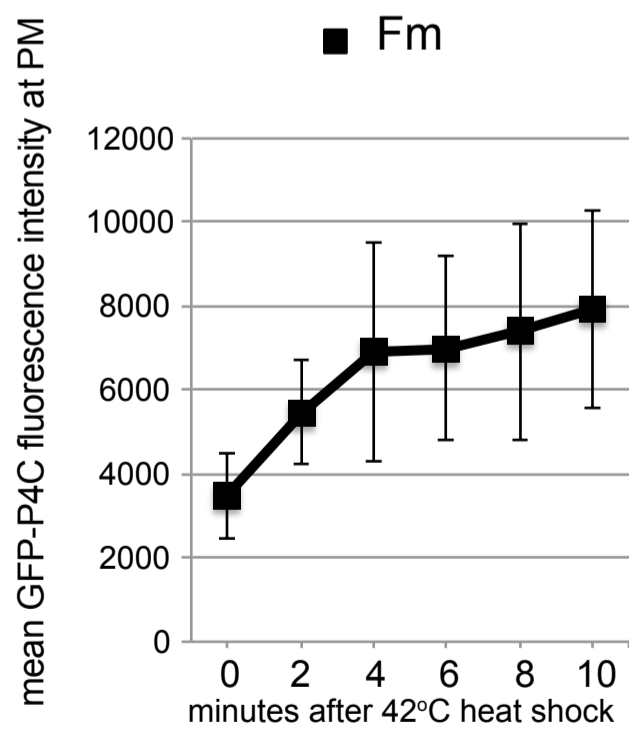

Supplement: Supplementary file 1 — Figure S1. PI4P distribution is regulated by growth conditions. (a) Cells expressing the PI4P reporter GFP-P4C (green) and a PM marker mCherry-2xPHPLCδ (magenta) were grown at 26 °C (left panels) and subjected to a heat shock for 10 min at 42 °C (right panels). Mother cells are indicated (m) and arrows point to GFP-P4C localization at the PM of mother cells at 42 °C. Scale bars, 5 μm. (b) Representative images of a time course of cells expressing the PI4P reporter GFP-P4C subjected to a heat shock at 42 °C. Cells were grown at 26 °C, immobilized on a 2% agarose pad mounted on a microscope slide. Arrowheads point to GFP-P4C localization at the PM of mother cells. Cells were imaged over time at 42 °C using a BIOPTECHS Objective Heater System. Scale bar, 5 μm. (c) Graph displays the mean Fd/Fm ratios of GFP-P4C fluorescence at 26 °C (t=0) and during heat shock at 42 °C for different time points (2 min intervals, see B). Error bars represent standard deviation. In total, 10 cells from two independent experiments were analyzed. (d) Graph shows the mean GFP-P4C fluorescence intensity at the mother cell PM (Fm) at 26 °C (t=0) and during heat shock at 42 °C at different time points (2 min intervals, see b and c). In total, 10 cells from two independent experiments were analyzed. [file 12915_2020_758_MOESM1_ESM.pdf]

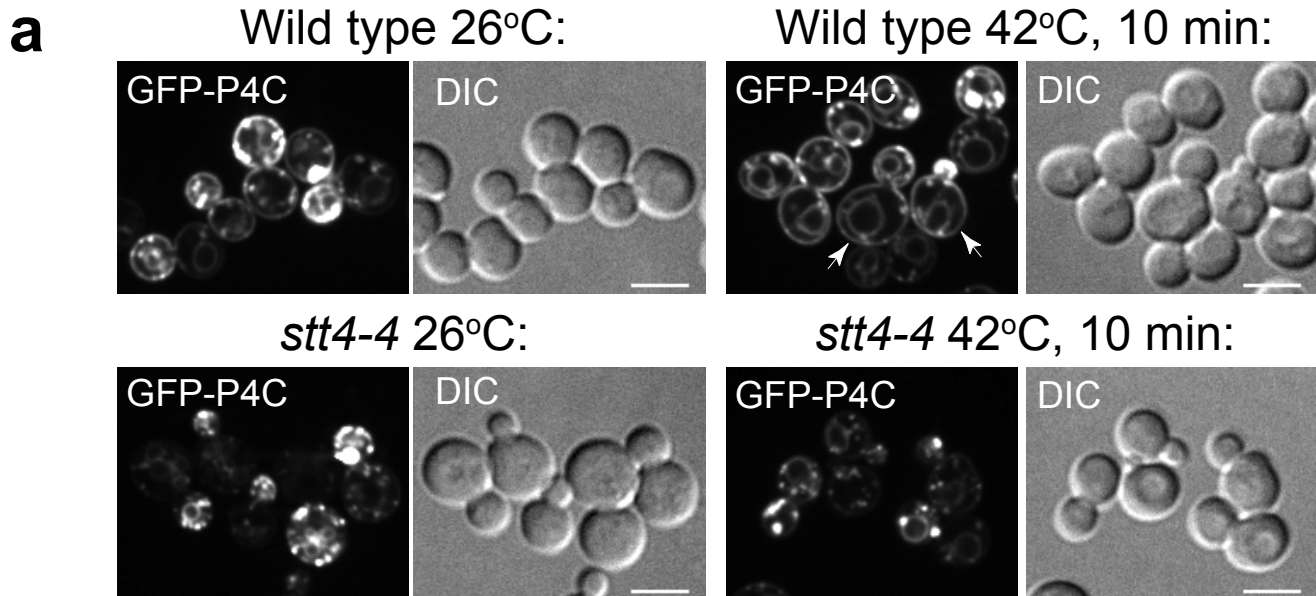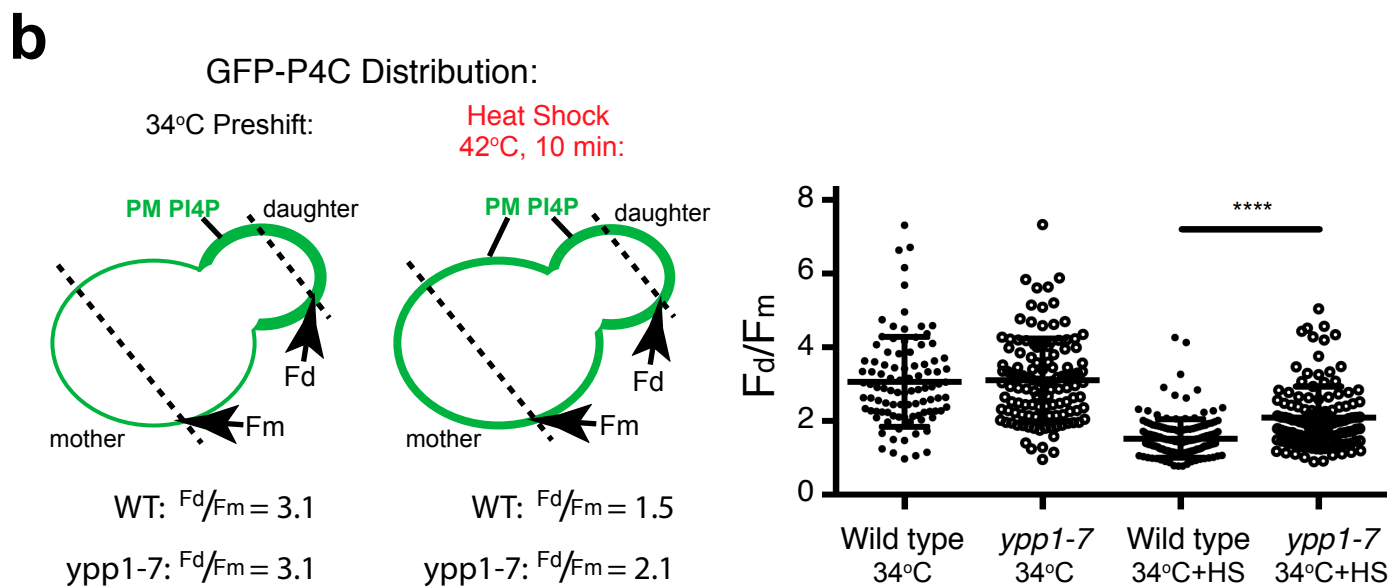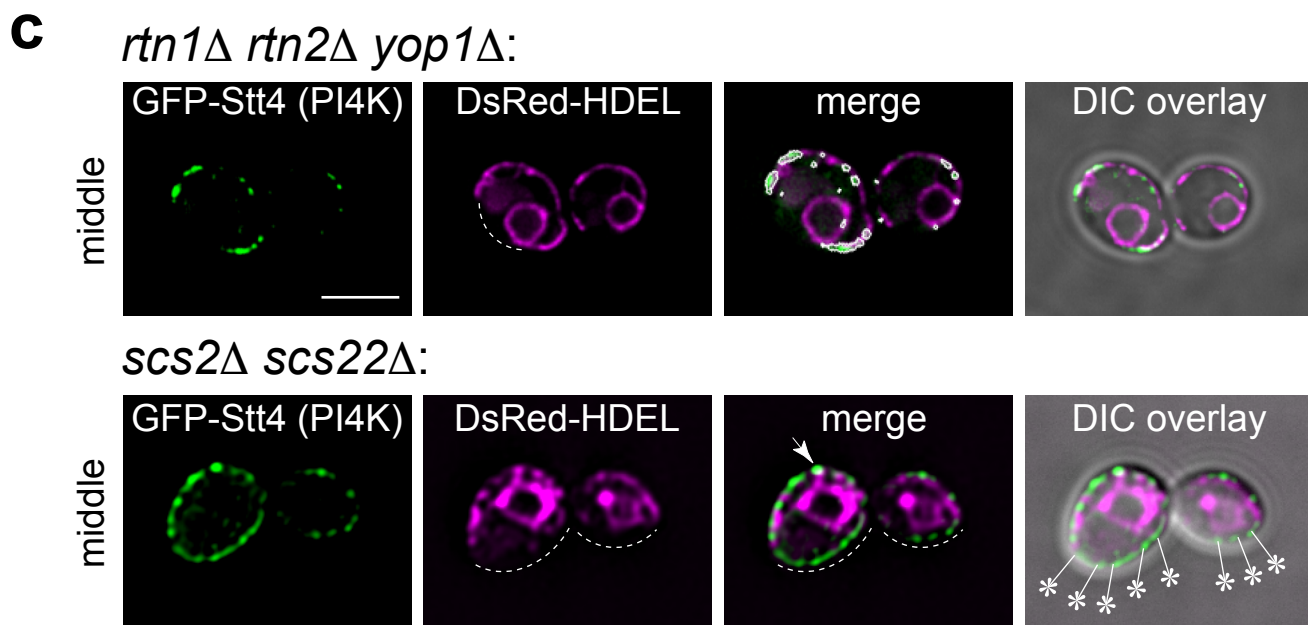

Supplement: Supplementary file 4 — Figure S2. Stt4 PIK patches localize to ER-PM contact sites and contribute to heat stress-induced PI4P signaling. (a) The Stt4 PI4K generates PI4P at the PM. Wild type cells (upper panel) and temperature conditional stt4-4 cells (lower panel) expressing the PI4P reporter GFP-P4C grown at 26 °C and after heat shock at 42 °C. Arrows point to GFP-P4C localization at the PM of mother cells at 42 °C. Scale bars, 5 μm. (b) Schematic representation of the method used to measure PM GFP-P4C fluorescence intensities at 34 °C and after 42 °C heat shock (left). Briefly, line scans were applied through both daughter and mother cells using Fiji and the peak values corresponding to the GFP-P4C fluorescence intensity at the PM in the daughter (Fd) and mother cell (Fm) were recorded to calculate Fd/Fm ratios. Graph shows the Fd/Fm ratio of individual cells at 34 °C and after a 10 min heat shock at 42 °C. Total number of cells analyzed: wild type 34 °C n=97, wild type 10min 42 °C n=160, ypp1-7 34 °C n=118, ypp1-7 10min 42 °C n=123. Mean values and standard deviations from four independent experiments are shown (one-way ANOVA, ****p<0.0001). (c) Localization of GFP-Stt4 (green) and the ER marker DsRed-HDEL (magenta) in rtn1∆ rtn2∆ yop1∆ mutant cells (upper panel) or scs2∆ scs22∆ (lower panel) mutant cells. In rtn1∆ rtn2∆ yop1∆ mutant cells, Stt4 PIK patches (outlined in white) are associated with the cortical ER (magenta) and are absent from ER-free PM zones (dashed lines). In scs2∆ scs22∆ mutant cells, Stt4 PIK patches (marked by asterisks) are found in ER-free PM zones (dashed lines). The arrow points to a PIK patch associated with the cortical ER (merge) in scs2∆ scs22∆ mutant cells. Thus, Stt4 may also localize to Scs2/22-independent ER-PM contacts, consistent with distinct Stt4 complexes (I and II; see Figure 2a). Scale bar, 4 μm. [file 12915_2020_758_MOESM4_ESM.pdf]

**a**

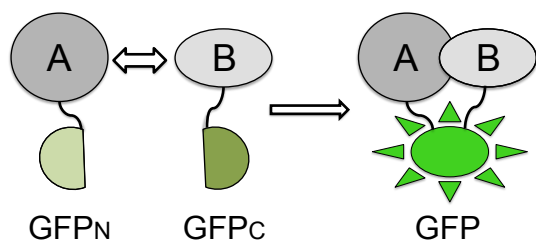

**b**

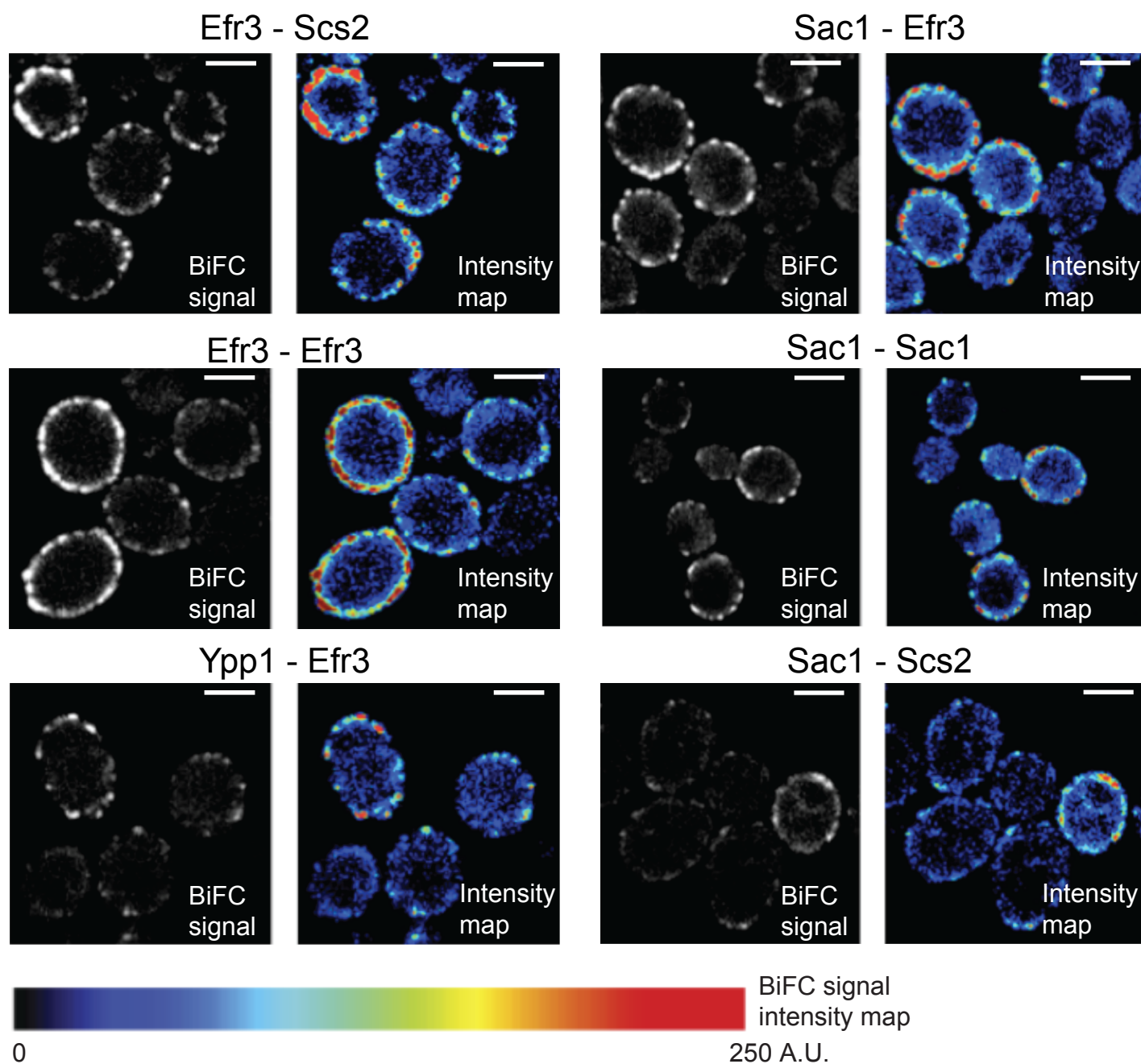

Supplement: Supplementary file 8 — Figure S3. ER-localized Scs2 interacts with the PIK patch subunit Efr3 at the PM and the PI4P phosphatase Sac1 in the ER. (a) Cartoon displaying the principle of BiFC using the split GFP assay. The N-terminal half of GFP (GFPN) and the C-terminal half of GFP (GFPC) only form a fluorescent GFP when brought into spatial proximity if their fusion partners, protein A and protein B, interact with each other. (b) Protein-protein interactions between Efr3, Scs2, Sac1, and Ypp1 as detected by the split GFP BiFC assay. In each case, GFPN is fused to the protein on listed on the left and GFPC is fused to the protein on listed on the right. In the Efr3-Sac1 pairing, for example, cells express Efr3-GFPN and Sac1-GFPC. The pseudo-colored images (intensity maps) indicate the scale of specific interactions (blue, moderate; red, strong). Scale bars, 3 μm [file 12915_2020_758_MOESM8_ESM.pdf]

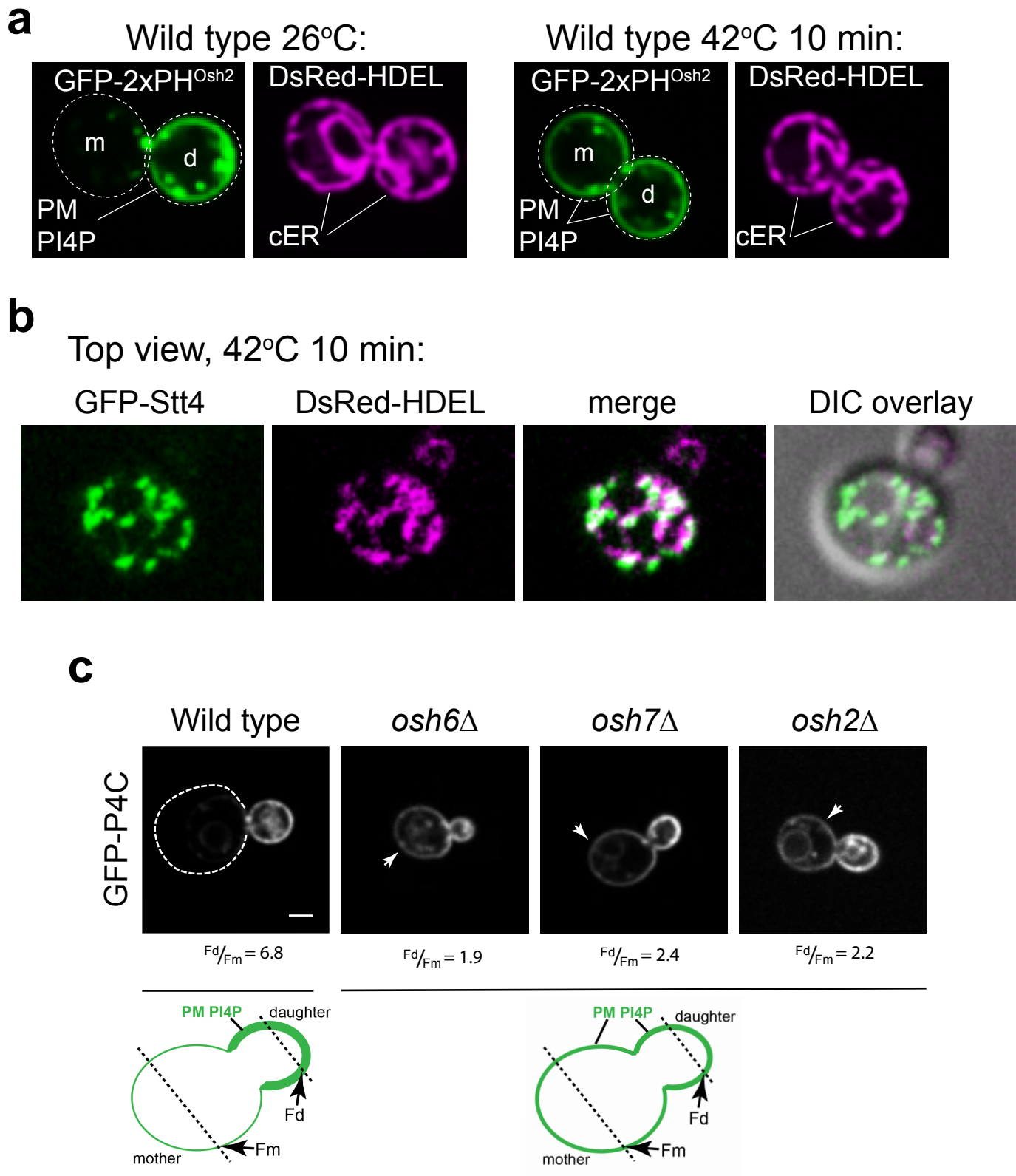

Supplement: Supplementary file 11 — Figure S4. Heat shock does not disrupt the cortical ER network, Stt4 localization at ER-PM contacts, or the cortical localization of Osh2 and Osh7. (a) Wild type cells expressing the PI4P reporter GFP-2xPHOsh2 (green) and the ER marker DsRed-HDEL (magenta) were grown at 26 °C (left panels) and subjected to a heat shock at 42 °C for 10 minutes (right panels). Cortical ER is present and observed under both conditions. (b) Top view images of a cell expressing GFP-Stt4 (green) and the ER marker DsRed-HDEL (magenta) after a heat shock for 10 min at 42 °C. (c) Representative examples of wild type, osh6∆, osh7∆, and osh2∆ cells expressing the PI4P reporter GFP-P4C grown at 26 °C to mid-log phase. GFP-P4C fluorescence intensities at the plasma membrane of daughter cells (Fd) and mother cells were measured as indicated and corresponding Fd/Fm ratios for the cells shown are indicated under each image. The periphery of the wild type mother cell is indicated (dashed white line). Arrows point to the PI4P reporter at the PM in mother cells. Scale bar, 2 μm. [file 12915_2020_758_MOESM11_ESM.pdf]

# Omnus *et al.*, Figure S6

**a**

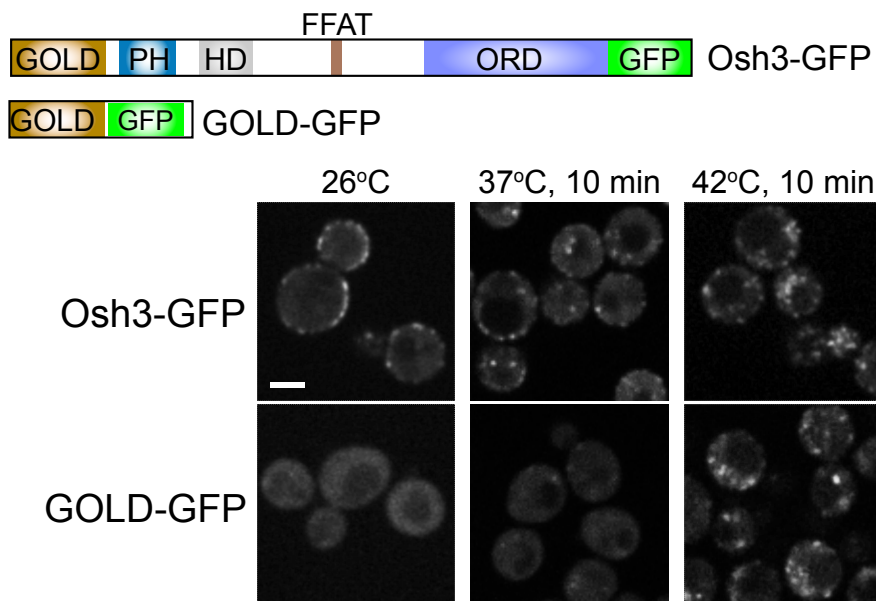

**b**

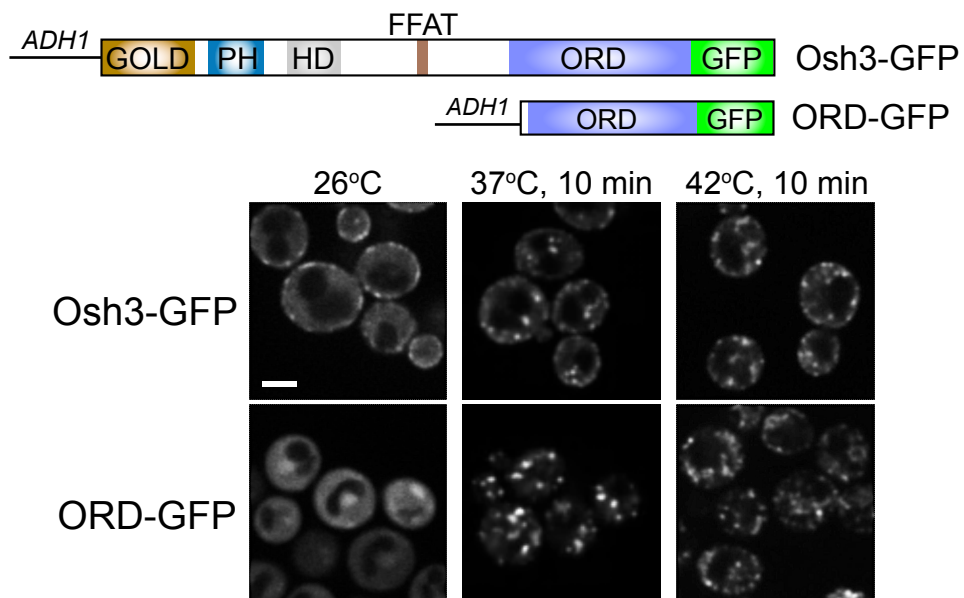

**c**

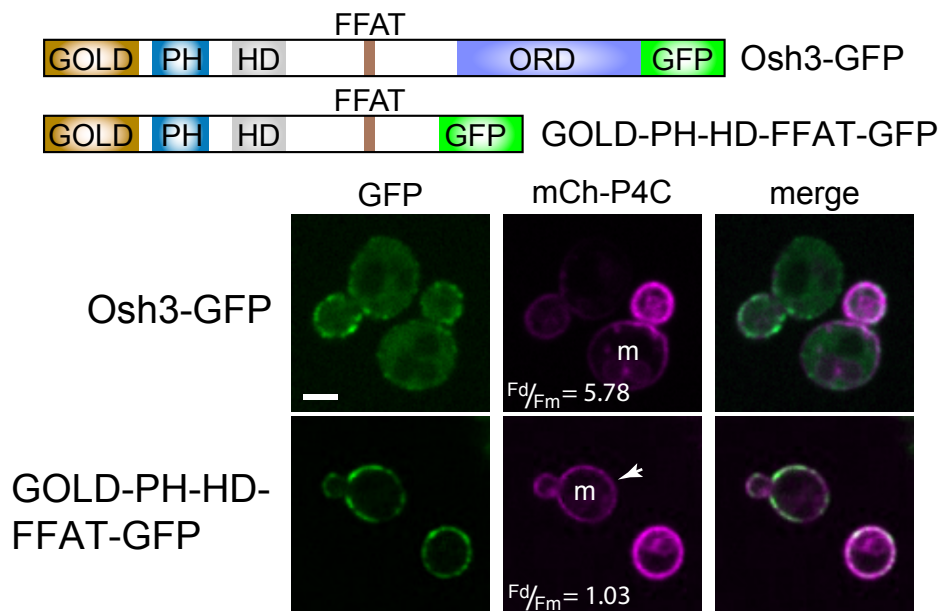

Supplement: Supplementary file 19 — Figure S6. The Osh3 GOLD and ORD regions aggregate upon brief heat stress conditions. (a) Schematic representations and cellular localization of full length Osh3-GFP and the C-terminal Osh3 truncation protein GOLD-GFP. The truncation was performed by homologous recombination and both proteins were expressed from the OSH3 promoter. Abbreviations shown are: GOLD, Golgi dynamics domain; PH, pleckstrin homology domain; HD, helical domain; FFAT, two phenyalanines in an acidic tract; ORD, OSBP-related domain; GFP, green fluorescent protein. Cells expressing full length Osh3-GFP or GOLD-GFP were grown at 26 °C and then shifted to 37 °C or 42 °C for 10 min prior to imaging by spinning disk confocal microscopy. Scale bar, 2 μm. (b) Schematic representations and cellular localization of full length Osh3-GFP and the N-terminal Osh3 truncation protein ORD-GFP. The truncation was performed by homologous recombination and both proteins were expressed from the ADH1 promoter. Abbreviations are the same as in Figure S6a. Cells expressing full length Osh3-GFP or ORD-GFP were grown at 26 °C and then shifted to 37 °C or 42 °C for 10 min prior to imaging by spinning disk confocal microscopy. Scale bar, 2 μm. (c) Localization of the PI4P reporter mCherry-P4C FLARE (magenta) in cells expressing either full length Osh3-GFP (green) or a truncated Osh3 protein lacking the ORD domain (GOLD-PH-HD-FFAT-GFP, green). The truncation was performed by homologous recombination and both proteins were expressed from the OSH3 promoter. Corresponding Fd/Fm ratios for the cells shown are indicated in each image. Arrow points to PI4P at the PM in a mother cell. Abbreviations are the same as in Figure S6a. Cells were grown at 26 °C to mid-log phase prior to imaging by spinning disk confocal microscopy. Scale bar, 2 μm. [file 12915_2020_758_MOESM19_ESM.pdf]

**a**

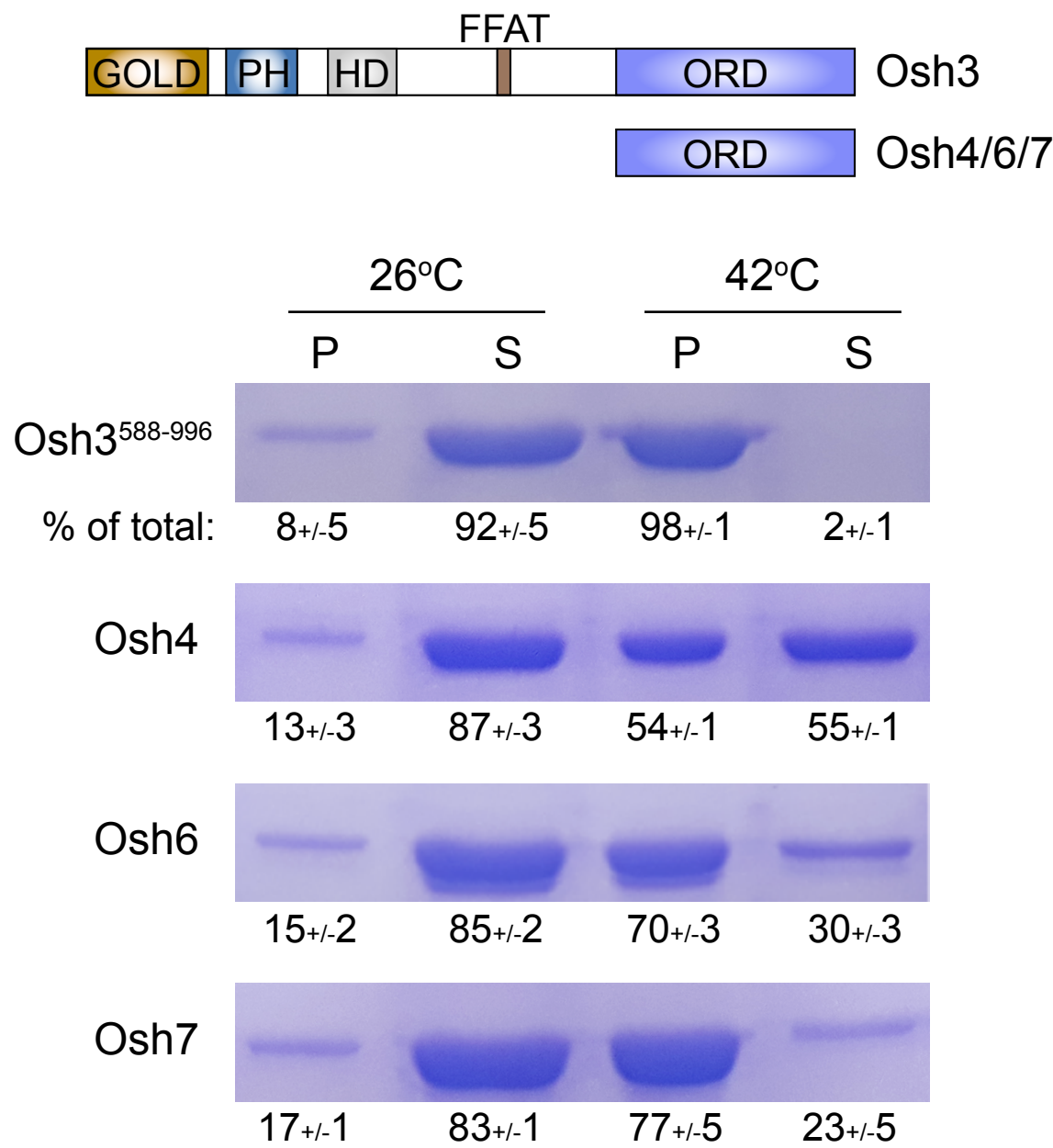

**b**

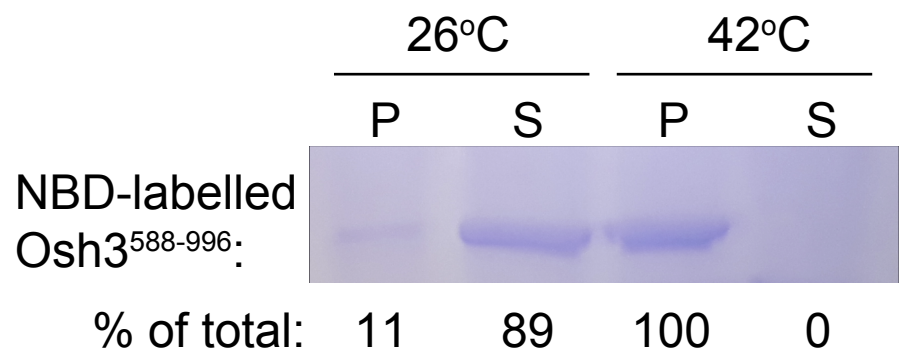

Supplement: Supplementary file 21 — Figure S7. The PI4P-binding ORD region of Osh proteins is heat sensitive in vitro. (a) (Top panel) Schematic representations of full length Osh3, Osh4, Osh6 and Osh7. Abbreviations: GOLD, Golgi dynamics domain; PH, pleckstrin homology domain; HD, helical domain; FFAT, two phenyalanines in an acidic tract; ORD, OSBP-related domain. (Bottom panels) The ORD region of Osh proteins sediments at elevated temperature. Purified Osh3588–996, his-Osh4, Osh6 and his-Osh7 were subjected to incubation at the indicated temperatures for 10 min prior to ultracentrifugation. P, pellet fraction; S, supernatant fraction. Quantitations of fractions are the averages and standard deviations from three independent experiments. (b) NBD-labelled Osh3588-996 sediments at elevated temperature. Purified NBD-labelled Osh3588-996 (see Figure 7) was subjected to incubation at the indicated temperatures for 10min prior to ultracentrifugation. P, pellet fraction; S, supernatant fraction. [file 12915_2020_758_MOESM21_ESM.pdf]

Omnus *et al.*, Figure S8

**a**

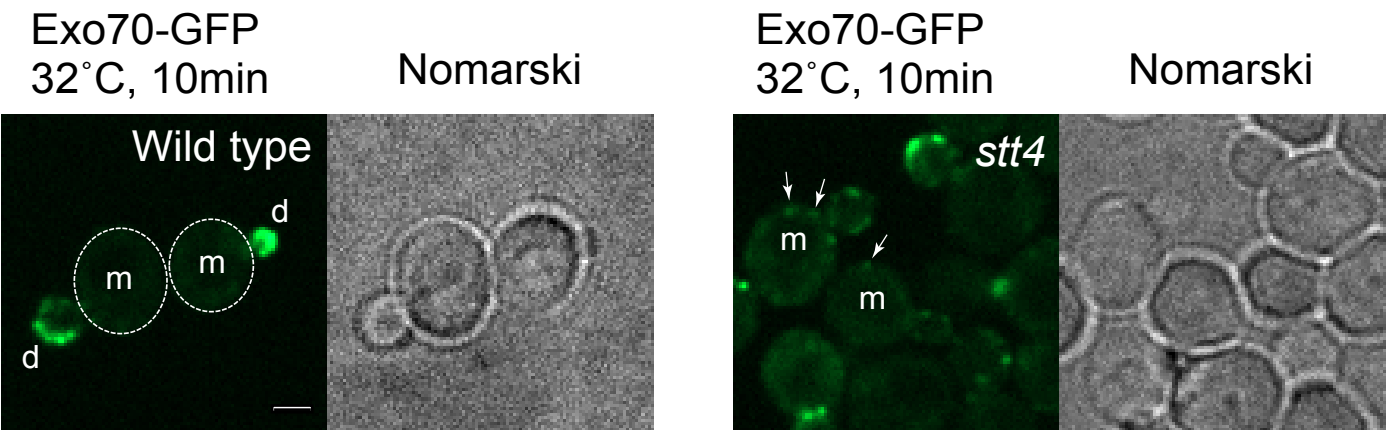

**b**

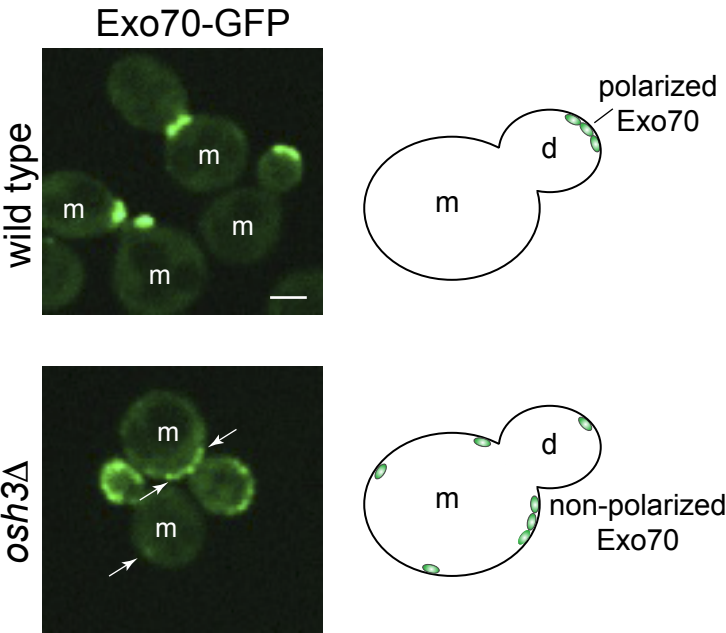

**c**

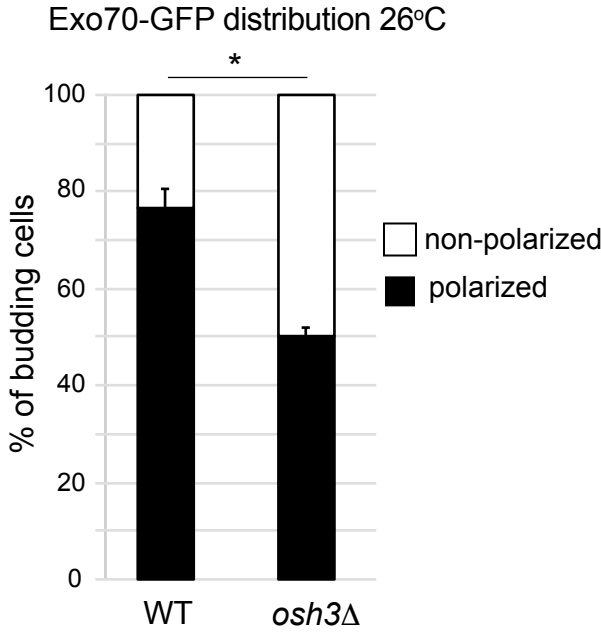

**d**

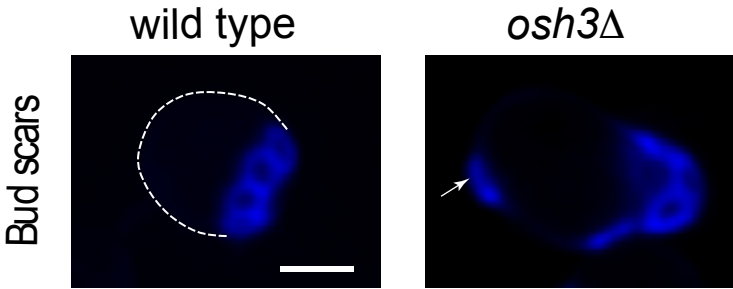

Supplement: Supplementary file 23 — Figure S8. Osh3 regulates the polarized localization of the exocyst subunit Exo70 and polarized secretion of the chitin synthase Chs3. (a) Wild type and temperature conditional stt4-4 mutant cells expressing Exo70-GFP at 26 °C were grown to log phase at 26 °C, shifted 10 min at 32 °C, and then imaged by spinning disk confocal microscopy. Representative confocal sections showing Exo70-GFP localization in wild type and stt4-4 mutant cells and corresponding Nomarski images are provided. Arrows point to non-polarized Exo70-GFP foci in stt4-4 mother cells. Mother (m) and daughter (d) cells are indicated. Scale bar, 2 μm. (b) Exponentially growing wild type or osh3∆ cells expressing Exo70-GFP at 26 °C were imaged by spinning disk confocal microscopy. Representative confocal sections showing Exo70-GFP localization in wild type and osh3∆ mutant cells are provided. Arrows point to non-polarized Exo70-GFP foci in mother cells lacking Osh3 (osh3∆). Mother (m) and daughter (d) cells are indicated. Scale bar, 2 μm. (c) Quantitative analysis of Exo70-GFP polarization in small-budded cells at 26 °C. Total number of cells analyzed: wild type 26 °C n=212, osh3∆ 26 °C n=208. The graph shows the means and standard deviations from three independent experiments (t test, *p< 0.015). (d) Exponentially growing wild type and osh3∆ cells were stained with calcofluor white (a dye that stains cell wall chitin enriched at sites of cell division known as bud scars). Representative Z projections showing bud scar distribution are provided. Arrow shows a non-polarized bud scar in an osh3∆ mutant cell. Scale bar, 2 μm. [file 12915_2020_758_MOESM23_ESM.pdf]
