# Supplementary material for: A heat-sensitive Osh protein controls PI4P polarity
Source: BMC Biol. 2020 Mar 13;18:28. doi: 10.1186/s12915-020-0758-x (PMC7071650; doi:10.1186/s12915-020-0758-x)

Omnus *et al.*, Figure S5

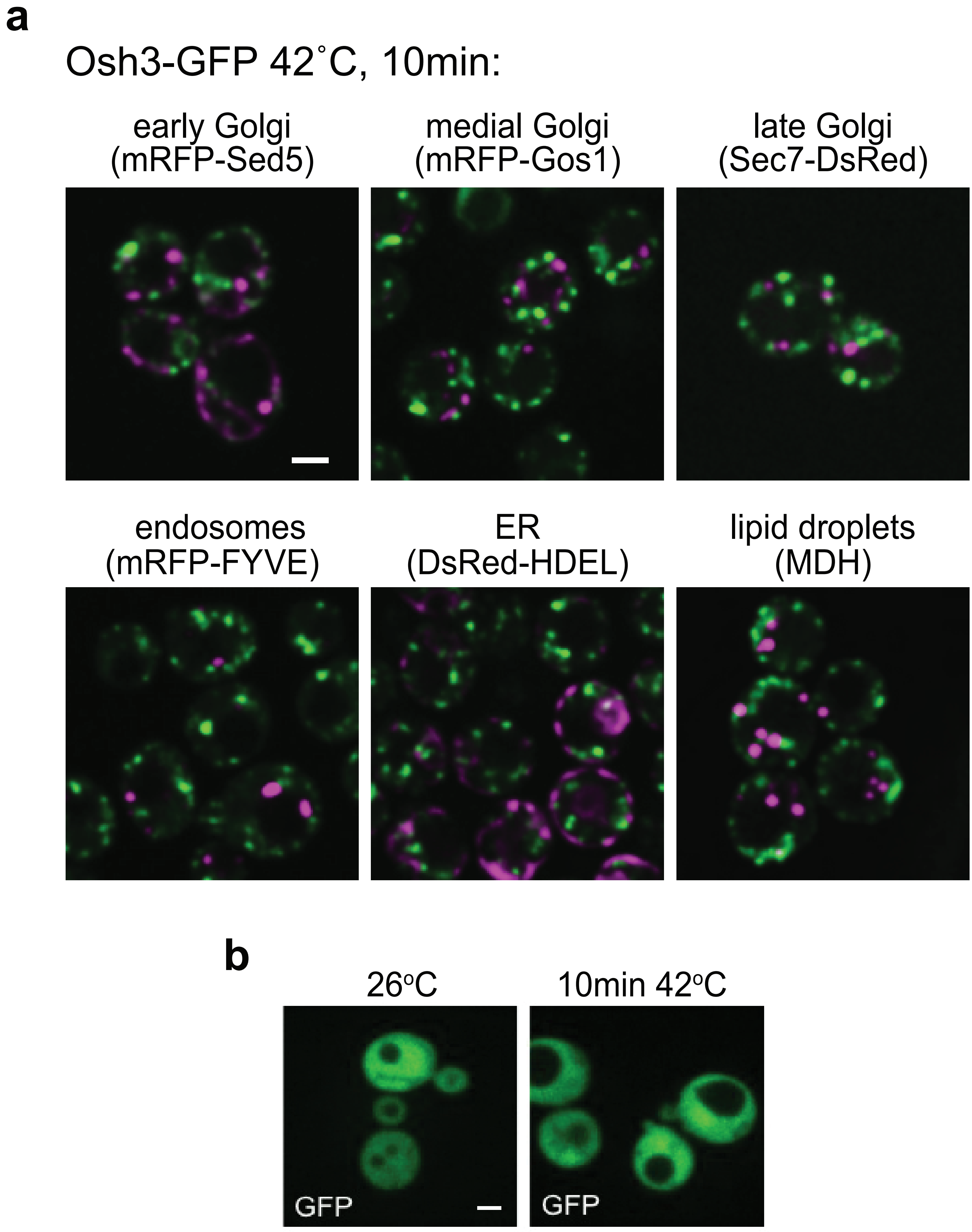

Supplement: Supplementary file 15 — Figure S5. Heat stress-induced Osh3-GFP aggregates do not co-localize with membrane-bound organelles. (a) Cells expressing Osh3-GFP (green) under its endogenous promoter were co-labelled with established markers of various different organelles (magenta): mRFP-Sed5 (early Golgi compartments), mRFP-Gos1 (medial Golgi compartments), Sec7-DsRed (late Golgi compartments), mRFP-FYVE (PI3P-containing endosomes), DsRed-HDEL (endoplasmic reticulum; ER) and MDH (lipid droplets). Cells were grown at 26 °C and then shifted 10 min at 42 °C prior to imaging. Scale bar, 2 μm. (b) Wild type cells expressing GFP were grown at 26 °C (left panel) and subjected to a heat shock for 10 min at 42 °C (right panel). Scale bar, 3 μm. (PDF 638 kb) [file 12915_2020_758_MOESM15_ESM.pdf]
