# Supplementary material for: A heat-sensitive Osh protein controls PI4P polarity
Source: BMC Biol. 2020 Mar 13;18:28. doi: 10.1186/s12915-020-0758-x (PMC7071650; doi:10.1186/s12915-020-0758-x)
Supplement: Supplementary file 18 — Osh3 Localization Dataset [file 12915_2020_758_MOESM18_ESM.pdf]

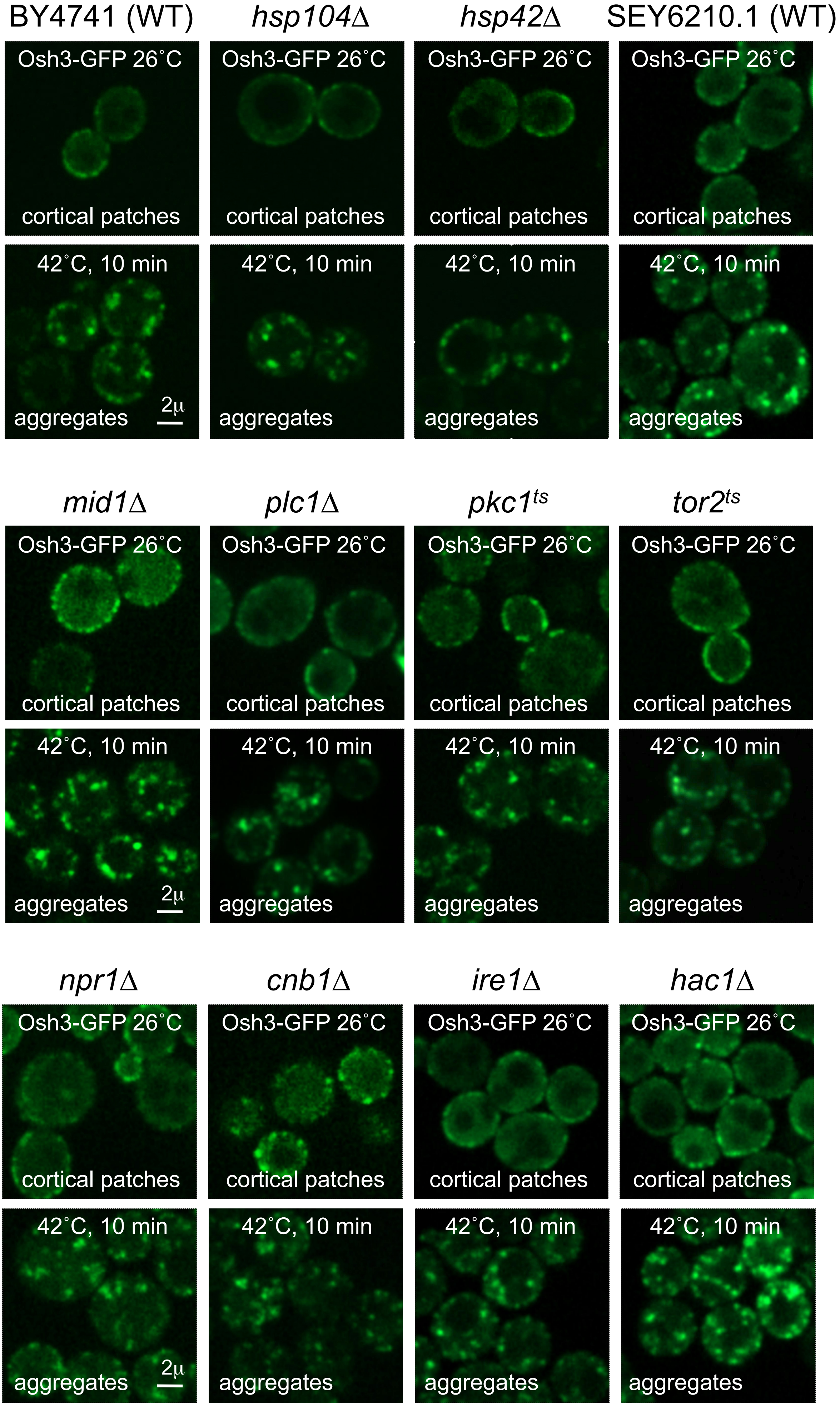
BY4741 (WT)
hsp104Δ
hsp42Δ
SEY6210.1 (WT)
Osh3-GFP 26˚C
cortical patches
42˚C, 10 min
aggregates
2μ
mid1Δ
plc1Δ
pkc1ts
tor2ts
npr1Δ
cnb1Δ
ire1Δ
hac1Δ
