# Supplementary material for: A heat-sensitive Osh protein controls PI4P polarity
Source: BMC Biol. 2020 Mar 13;18:28. doi: 10.1186/s12915-020-0758-x (PMC7071650; doi:10.1186/s12915-020-0758-x)
Supplement: Supplementary file 26 — Table 1 [file 12915_2020_758_MOESM26_ESM.docx]

| **Table 1. *Saccharomyces cerevisiae* strains used in this study.** | | |
| --- | --- | --- |
| **Strain** | **Genotype** | **Reference* or Source** |
| SEY6210 | *MATα* *leu2-3,112 ura3-52 his3Δ200 trp1-Δ901*  *lys2-801 suc2Δ9* | [1] |
| SEY6210.1 | *MAT****a*** *leu2-3,112 ura3-52 his3Δ200 trp1-Δ901*  *lys2-801 suc2Δ9* | [1] |
| AAY102 | SEY6210 *stt4*Δ::*HIS3* harboring pRS415*stt4-4* | [2] |
| AAY182 | SEY6210 *sfk1*Δ::*HIS3* | [3] |
| DOY10 | SEY6210 *DsRed-HDEL::LEU2* | This study |
| DOY11 | SEY6210 *DsRed-HDEL::URA3* | This study |
| DOY12 | SEY6210 *YPP1-GFP*::*TRP1 DsRed-HDEL::URA3* | This study |
| DOY13 | SEY6210 *stt4*Δ::*HIS3* *DsRed-HDEL::URA3* harboring pRS415-GFP*-STT4* | This study |
| YCS507 | SEY6210 *rtn1*Δ*::TRP1 rtn2*Δ*::HIS3MX6 yop1*Δ*::TRP1* | [4] |
| YCS557 | YCS507 *stt4*Δ::*HIS3* harboring pRS415-GFP*-STT4* | This study |
| DBY356 | SEY6210.1 *scs2*Δ*::TRP1 scs22*Δ*::HIS3MX6* | [4] |
| HHY5 | SEY6210 *EFR3-GFP_C_*::*HIS3MX6* | This study |
| HHY6 | SEY6210.1 *EFR3-GFP_N_*::*TRP1* | This study |
| YCS2341 | SEY6210.1 *EFR3ΔFFAT-GFP_N_*::*TRP1* | This study |
| HHY7 | SEY6210 *SAC1-GFP_C_*::*HIS3MX6* | This study |
| HHY8 | SEY6210.1 *SAC1-GFP_N_*::*TRP1* | This study |
| HHY9 | SEY6210 *YPP1-GFP_C_*::*HIS3MX6* | This study |
| HHY10 | SEY6210.1 *YPP1-GFP_N_*::*TRP1* | This study |
| YCS473 | SEY6210 *OSH2-GFP*::*TRP1* | [4] |
| YCS484 | SEY6210 *OSH3-GFP*::*HIS3MX6* | [4] |
| YCS597 | SEY6210 *OSH7-GFP*::*HIS3MX6* | This study |
| JRY6202 | SEY6210 *osh3*Δ::*LYS2* | [5] |
| YCS2336 | SEY6210.1 *HSP104-mCherry*::*TRP1* | This study |
| YAC132 | SEY6210.1 *OSH3-GFP*::*NATMX6* | This study |
| YAC118 | SEY6210.1 *GOLD^Osh3^-GFP*::*NATMX6* | This study |
| YAC123 | SEY6210.1 p*ADH1*-*OSH3-GFP*::*NATMX6, HIS3MX6* | This study |
| YAC125 | SEY6210.1 p*ADH1*-*ORD^Osh3^-GFP*::*NATMX6, HIS3MX6* | This study |
| YCS247 | SEY6210 *pkc1*Δ::*LEU2* harboring YCp50-*pkc1^ts^* | [3] |
| YCS369 | SEY6210 *plc1*Δ::*LEU2* | This study |
| YCS384 | SEY6210 *cnb1*Δ::*HIS3MX6* | This study |
| YCS647 | SEY6210 *mid1*Δ::*HIS3MX6* | This study |
| YCS678 | SEY6210 *npr1*Δ::*HIS3MX6* | [6] |
| AAY2007 | SEY6210 *tor2*Δ::*HIS3MX6* harboring pRS415-*tor2-1* | [3] |
| YCS645 | SEY6210 *ire1*Δ::*TRP1* | [7] |
| YCS1856 | SEY6210 *hac1*Δ::*NATMX6* | [8] |
| BY4741 | *MAT***a** *his3Δ1 leu2Δ0 met15Δ0 ura3Δ0* | [9] |
|  | BY4741 *hsp104*Δ*::kanMX6* | [9-11] |
|  | BY4741 *hsp42*Δ*::kanMX6* | [9-11] |
|  | BY4741 *sfk1*Δ*::kanMX6* | [9-11] |
| DBY16 | BY4741 *ypp1*Δ*::HIS3MX6* carrying pRS415-*ypp1-7* | [12] |
| JRY6201 | SEY6210 *osh6*∆::*LEU2* | [5] |
| JRY6200 | SEY6210 *osh7*∆::*HIS3* | [5] |
| JRY6203 | SEY6210 *osh2*∆::*URA3* | [5] |

***Note: References for Table 1 are provided below and some are also cited (with different numbering) in the main article.**

**References**

1. Robinson JS, Klionsky DJ, Banta LM, Emr SD: Protein sorting in Saccharomyces cerevisiae: isolation of mutants defective in the delivery and processing of multiple vacuolar hydrolases. *Molecular and cellular biology* 1988, 8(11):4936-4948.

2. Audhya A, Foti M, Emr SD: Distinct roles for the yeast phosphatidylinositol 4-kinases, Stt4p and Pik1p, in secretion, cell growth, and organelle membrane dynamics. *Mol Biol Cell* 2000, 11(8):2673-2689.

3. Audhya A, Emr SD: Stt4 PI 4-kinase localizes to the plasma membrane and functions in the Pkc1-mediated MAP kinase cascade. *Dev Cell* 2002, 2(5):593-605.

4. Stefan CJ, Manford AG, Baird D, Yamada-Hanff J, Mao Y, Emr SD: Osh proteins regulate phosphoinositide metabolism at ER-plasma membrane contact sites. *Cell* 2011, 144(3):389-401.

5. Beh CT, Cool L, Phillips J, Rine J: Overlapping functions of the yeast oxysterol-binding protein homologues. *Genetics* 2001, 157(3):1117-1140.

6. MacGurn JA, Hsu PC, Smolka MB, Emr SD: TORC1 regulates endocytosis via Npr1-mediated phosphoinhibition of a ubiquitin ligase adaptor. *Cell* 2011, 147(5):1104-1117.

7. Zhao Y, Macgurn JA, Liu M, Emr S: The ART-Rsp5 ubiquitin ligase network comprises a plasma membrane quality control system that protects yeast cells from proteotoxic stress. *Elife* 2013, 2:e00459.

8. Manford AG, Stefan CJ, Yuan HL, Macgurn JA, Emr SD: ER-to-plasma membrane tethering proteins regulate cell signaling and ER morphology. *Dev Cell* 2012, 23(6):1129-1140.

9. Brachmann CB, Davies A, Cost GJ, Caputo E, Li J, Hieter P, Boeke JD: Designer deletion strains derived from Saccharomyces cerevisiae S288C: a useful set of strains and plasmids for PCR-mediated gene disruption and other applications. *Yeast* 1998, 14(2):115-132.

10. Winzeler EA, Shoemaker DD, Astromoff A, Liang H, Anderson K, Andre B, Bangham R, Benito R, Boeke JD, Bussey H *et al*: Functional characterization of the S. cerevisiae genome by gene deletion and parallel analysis. *Science* 1999, 285(5429):901-906.

11. Giaever G, Chu AM, Ni L, Connelly C, Riles L, Veronneau S, Dow S, Lucau-Danila A, Anderson K, Andre B *et al*: Functional profiling of the Saccharomyces cerevisiae genome. *Nature* 2002, 418(6896):387-391.

12. Baird D, Stefan C, Audhya A, Weys S, Emr SD: Assembly of the PtdIns 4-kinase Stt4 complex at the plasma membrane requires Ypp1 and Efr3. *J Cell Biol* 2008, 183(6):1061-1074.
