# Supplementary material for: A heat-sensitive Osh protein controls PI4P polarity
Source: BMC Biol. 2020 Mar 13;18:28. doi: 10.1186/s12915-020-0758-x (PMC7071650; doi:10.1186/s12915-020-0758-x)
Supplement: Supplementary file 27 — Table 2 [file 12915_2020_758_MOESM27_ESM.docx]

| **Table 2. Plasmids used in this study.** | | |
| --- | --- | --- |
| **Plasmid Name** | **Description** | **Reference* or Source** |
| pBP73G-P4C | pRS416-p*GPD*-GFP-P4C^SidC^ | [1] |
| pYL95 | pRS415-pGPD-mCherry-2xPH^PLC δ^ | [2] |
| pCS276 | pRS426-p*PRC1*-GFP-2xPH^Osh2^ | [3] |
| pCS357 | pRS424-p*PRC1*-GFP-2xPH^Osh2^ | [3] |
| pCS403 | pRS415-pGPD-mCherry-2xPH^Osh2^ | [4] |
| pCS1978 | pRS305-DsRed-HDEL | This study |
| pCS1970 | pRS306-DsRed-HDEL | This study |
|  | pRS416-DsRed-HDEL | [5] |
|  | pRS415-GFP-*STT4* | [6] |
|  | pRS415-*stt4-4* | [7] |
|  | pRS415-GFP_C_-*SCS2* | This study |
|  | pRS414-*OSH3*-GFP | [3] |
|  | pRS415-*OSH3*-GFP | [3] |
|  | pGEX6P1-Osh3^588-996^ | [3] |
| pCS1981 | pFA6a-GFP_C_::*HISMX6* | This study |
| pCS1980 | pFA6a-GFP_N_::*TRP1* | This study |
| pG365 | pRS304-*EXO70-*GFP::*TRP1* | [8] |
|  | pRS314-mRFP-*SED5* | Prof. Scott Emr |
|  | pRS314-mRFP-*GOS1* | Prof. Scott Emr |
|  | pRS316-*SEC*7-DsRed | [9] |
|  | pRS416-mRFP-FYVE | [10] |
|  | pRS416-*CHS3*-GFP | [11] |
| pGO-GFP | pRS426-GFP | [12] |
|  | pRSETB-Osh4 | [3] |
| pCS2722 | pRSETB-Osh7 | This study |
| pCS2735 | pGEX6P1-Osh6 | This study |

***Note: References for Table 2 are provided below and some are also cited (with different numbering) in the main article.**

**References**

1. Luo X, Wasilko DJ, Liu Y, Sun J, Wu X, Luo ZQ, Mao Y: Structure of the Legionella Virulence Factor, SidC Reveals a Unique PI(4)P-Specific Binding Domain Essential for Its Targeting to the Bacterial Phagosome. *PLoS Pathog* 2015, 11(6):e1004965.

2. Ling Y, Stefan CJ, Macgurn JA, Audhya A, Emr SD: The dual PH domain protein Opy1 functions as a sensor and modulator of PtdIns(4,5)P(2) synthesis. *The EMBO journal* 2012, 31(13):2882-2894.

3. Stefan CJ, Manford AG, Baird D, Yamada-Hanff J, Mao Y, Emr SD: Osh proteins regulate phosphoinositide metabolism at ER-plasma membrane contact sites. *Cell* 2011, 144(3):389-401.

4. Omnus DJ, Manford AG, Bader JM, Emr SD, Stefan CJ: Phosphoinositide kinase signaling controls ER-PM cross-talk. *Mol Biol Cell* 2016, 27(7):1170-1180.

5. Audhya A, Emr SD: Regulation of PI4,5P2 synthesis by nuclear-cytoplasmic shuttling of the Mss4 lipid kinase. *The EMBO journal* 2003, 22(16):4223-4236.

6. Audhya A, Emr SD: Stt4 PI 4-kinase localizes to the plasma membrane and functions in the Pkc1-mediated MAP kinase cascade. *Dev Cell* 2002, 2(5):593-605.

7. Audhya A, Foti M, Emr SD: Distinct roles for the yeast phosphatidylinositol 4-kinases, Stt4p and Pik1p, in secretion, cell growth, and organelle membrane dynamics. *Mol Biol Cell* 2000, 11(8):2673-2689.

8. He B, Xi F, Zhang X, Zhang J, Guo W: Exo70 interacts with phospholipids and mediates the targeting of the exocyst to the plasma membrane. *The EMBO journal* 2007, 26(18):4053-4065.

9. Calero M, Chen CZ, Zhu W, Winand N, Havas KA, Gilbert PM, Burd CG, Collins RN: Dual prenylation is required for Rab protein localization and function. *Mol Biol Cell* 2003, 14(5):1852-1867.

10. Rue SM, Mattei S, Saksena S, Emr SD: Novel Ist1-Did2 complex functions at a late step in multivesicular body sorting. *Mol Biol Cell* 2008, 19(2):475-484.

11. Valdivia RH, Schekman R: The yeasts Rho1p and Pkc1p regulate the transport of chitin synthase III (Chs3p) from internal stores to the plasma membrane. *Proceedings of the National Academy of Sciences of the United States of America* 2003, 100(18):10287-10292.

12. Odorizzi G, Babst M, Emr SD: Fab1p PtdIns(3)P 5-kinase function essential for protein sorting in the multivesicular body. *Cell* 1998, 95(6):847-858.
